# Supplementary material for: Access to weight reduction interventions for overweight and obese patients in UK primary care: population-based cohort study
Source: BMJ Open. 2015 Jan 5;5(1):e006642. doi: 10.1136/bmjopen-2014-006642 (PMC4316417; doi:10.1136/bmjopen-2014-006642)
Supplement: Author's manuscript [file bmjopen-2014-006642.draft_revisions.pdf]

BMJ Open

Access to weight reduction interventions for overweight and obese patients in UK primary care. Population-based cohort study

Journal:	*BMJ Open*
Manuscript ID:	bmjopen-2014-006642
Article Type:	Research
Date Submitted by the Author:	15-Sep-2014
Complete List of Authors:	Booth, Helen; King's College London, Primary Care and Public Health Sciences<br>Prevost, A.; King's College London, Primary Care and Public Health Sciences<br>Gulliford, Martin; King's College London, Primary Care and Public Health Sciences
**Primary Subject Heading**:	General practice / Family practice
Secondary Subject Heading:	Public health
Keywords:	PRIMARY CARE, PUBLIC HEALTH, EPIDEMIOLOGY

SCHOLARONE™
Manuscripts

For peer review only

Access to weight reduction interventions for overweight and obese patients in UK primary care. Population-based cohort study

Running head: Obesity management in primary care

Article category: Original research

Authors:

Helen P. Booth[1], BSc MSc

A. Toby Prevost[1], BSc MSc PhD

Martin C. Gulliford[1], FFPH FRCP

[1]Department of Primary Care and Public Health Sciences, King's College London, UK

Corresponding author:

Miss Helen P. Booth, Research Associate, Department of Primary Care and Public Health Sciences, King's College London, 7th Floor Capital House, 42 Weston Street, London,

SE1 3QD. Tel: 020 7848 6104. Fax: 020 7848 6620 Email: helen.booth@kcl.ac.uk

References: 21

Tables: 5

Figures: 0

Word count: 2,261

Abstract word count: 283

Supplementary files: 0

Key words: Obesity, Overweight, Primary health care, General practice

For peer review only

ABSTRACT

Background: Two-thirds of UK adults are overweight or obese. Primary care services are the first point of access for clinical body weight management and care of obesity-related conditions.

Objective: This study aimed to investigate access to weight management interventions for overweight and obese patients in primary care.

Design: Population-based cohort study using primary care electronic health records

Methods: A cohort of overweight and obese patients aged 30 to 100 years was sampled from the Clinical Practice Research Datalink (CPRD). Body mass index (BMI) values for the cohort recorded between 2005 and 2012 were categorised using World Health Organisation (WHO) criteria. Utilisation of interventions for body weight management, including advice, referrals and prescription of anti-obesity drugs, were evaluated by BMI category.

Key results: Data were analysed for 91,413 patients, mean age 56 years, including 55,094 (60%) overweight and 36,319 (40%) obese, including 4,099 (5%) with morbid obesity. During the study period 90% of overweight patients had no weight management intervention recorded. Intervention was more frequent among obese patients, but 59% of patients with morbid obesity had no intervention recorded. Rates of intervention increased with BMI category. In morbid obesity, rates of intervention per 1,000 patient years were: advice, 60.2 (95% CI 51.8 to 70.4); referral, 75.7 (95% CI 69.5 to 82.6); and anti-obesity drugs 89.9 (95% CI 85.0 to 95.2). Weight management interventions were more often accessed by women, older patients, those with co-morbidity and those in deprivation. Follow-up of body weight subsequent to interventions was infrequent.

For peer review only - http://bmjopen.bmj.com/site/about/guidelines.xhtml

Conclusion: Limited evidence of weight management interventions in primary care electronic health records may result from poor recording of advice given, but may indicate a lack of access to appropriate body weight management interventions in primary care.

ARTICLE SUMMARY

Strengths and limitations of this study

- This study uses primary care electronic health records to investigate the use of weight management interventions in overweight and obese patients
- Lifestyle advice was the most commonly-used intervention in all but morbidly obese patients, where anti-obesity drugs were more frequent
- Patients had to have a BMI value recorded to be included in the study
- Clinicians may be giving advice for weight management but not recording it

For peer review only - http://bmjopen.bmj.com/site/about/guidelines.xhtml

BACKGROUND

Obesity is a leading cause of premature morbidity and mortality worldwide.(1) In the UK, a quarter of adults are obese and up to two thirds are overweight.(2) Primary care represents an important setting in which obese patients may access weight loss interventions. The main strategies for treating obesity are provision of lifestyle advice, referral for weight management, prescription of anti-obesity drugs and, in severe cases, referral for bariatric surgery. Clinical guidelines recommend a stepped approach to weight management depending on the severity of a patient's obesity and whether they have weight-related co-morbidities, with more intensive interventions offered as appropriate. Interventions should be agreed between the clinician and patient, and offered in conjunction with long-term follow-up and continuing care.(3)

Several studies have evaluated the effectiveness of primary care interventions for weight management in obesity, (4-8) but few studies have evaluated how overweight and obese patients are managed in primary care. A survey using data collected in 2000/1, before the UK national guidelines on obesity management were published,(9) found that a fifth of obese patients were offered dietary counselling, less than 5% a referral and 2% anti-obesity medications over an 18 month period. There is no more recent information on the use of interventions for the management of obesity in primary care.

Access to appropriate weight management interventions for overweight and obese patients in primary care is of increasing importance in the context of a national objective to establish a downward trend in obesity among UK adults by 2020.(10)

For peer review only

For peer review only - http://bmjopen.bmj.com/site/about/guidelines.xhtml

This study aimed to evaluate access in terms of recording and utilisation of weight management interventions for overweight and obesity using primary care electronic health records. Interventions were classified as lifestyle advice, referrals for weight management and prescription of anti-obesity drugs.

For peer review only

For peer review only - http://bmjopen.bmj.com/site/about/guidelines.xhtml

METHODS

Data source and cohort definition

A cohort of patients was selected from the Clinical Practice Research Datalink (CPRD), a database of longitudinal patient electronic medical records from UK primary care. CPRD is the largest primary care database in the world, and represents over 5% of the UK population with about 680 practices currently contributing research quality data.(11) The initial cohort comprised a random sample of approximately 300,000 patients who were registered for at least 12 months with a general practice contributing data to CPRD between the 1st January 2005 and 30th April 2012. Equal numbers of men and women were drawn from each year of the study without replacement. Patients were selected who had a body mass index (BMI) record indicating overweight or obesity during the study period. The study was approved by the CPRD Independent Scientific Advisory Committee (ISAC 07_054 and 14_056).

Exposure and outcome definitions

BMI was categorised using the World Health Organisation (WHO) categories: overweight (BMI 25-29.9kg/m^2), obese (BMI 30-34.9kg/m^2), severe obesity (BMI 35.0 to 39.9kg/m^2), morbid obesity (BMI≥40kg/m^2). Medical diagnoses of obesity were also noted. Morbidity status was ascertained based on the presence of eleven common conditions associated with obesity, including: type 2 diabetes, CHD, stroke, depression, osteoarthritis, back pain, joint problems, cancer, gallbladder disease, asthma and sleep apnoea. Smoking status and socioeconomic deprivation were also included as exposures. Socioeconomic deprivation was classified into quintiles using

For peer review only - http://bmjopen.bmj.com/site/about/guidelines.xhtml

the Index of Multiple Deprivation rank based on patient postcode. Data on deprivation were only available for patients registered at English practices.

Interventions for the management of body weight were identified using medical codes recorded in clinical and referral records, recorded health promotion advice, and prescriptions for anti-obesity drugs. Relevant referrals included those to community and hospital dieticians, and for exercise therapy. Prescriptions for three different anti-obesity drugs were included; two of these, sibutramine and rimonabant, have been removed from the UK market because of safety concerns.(12, 13) However, these drugs were in use during the time period investigated and so have been considered in this analysis. The only anti-obesity drug currently licensed in the UK, orlistat, was introduced in 1998 and has been available over the counter as well as by prescription since 2009.(14) Multiple prescriptions of anti-obesity drugs were considered to be a part of the same course of treatment if there was less than 6 months between prescriptions. For analysis, weight management interventions were classified into lifestyle advice, referrals for weight management and prescription of anti-obesity drugs.

Analysis

Person time was analysed following the index date; the first BMI record for overweight or obesity after the 1st January 2005. Patient baseline characteristics were tabulated. The proportion of patients who received weight management interventions over the study period was evaluated by BMI category. Time-to-event analysis was used to calculate the rate of intervention utilisation by BMI category and to investigate variables associated with intervention using a multiple-failure

For peer review only - http://bmjopen.bmj.com/site/about/guidelines.xhtml

multivariable Cox proportional hazards regression model with unordered events.(15) Variation in the use of weight management interventions by GP practice was investigated by calculating summary statistics for patients receiving any intervention in the year following the index date. Change in weight from baseline after the implementation of each type of intervention was calculated for up to 5 years of follow-up.

For peer review only

RESULTS

Data were analysed for 91,413 patients, with mean age 56 years, including 55,094 (60%) overweight and 36,319 (40%).or obese, including 4,099 (5%) with morbid obesity. Mean age in men and women was 56 years. Patient characteristics on entry to the study are presented in Table 1. At the index date most patients were overweight (63.9% of men and 56.2% of women); 2.9% of men and 6.3% of women were morbidly obese. A diagnostic code indicating obesity was recorded for 3.9% of male patients and 6.5% of females. A higher proportion of women were non-smokers, while men were more likely to be former or current smokers.

For peer review only

For peer review only - http://bmjopen.bmj.com/site/about/guidelines.xhtml

Table 1: Characteristics of overweight and obese patients. Figures are frequencies (column percent).

	Men (48,413)	Women (43,000)
**Mean age (SD)**	55.6 (13.9)	56.4 (15.0)
**BMI category (Kg/m2)**		
Overweight (BMI 25-29.9)	30,950 (63.9)	24,144 (56.2)
Obese (BMI 30-34.9)	12,711 (26.3)	11,364 (26.4)
Severe obesity (BMI 35-39.9)	3,368 (7.0)	4,777 (11.1)
Morbid obesity (BMI ≥40)	1,384 (2.9)	2,715 (6.3)
**Medical code for obesity**	1,876 (3.9)	2,810 (6.5)
**Number of morbidities**		
0	14,810 (30.6)	9,635 (22.4)
1	14,988 (31.0)	11,919 (27.7)
2	10,323 (21.3)	10,237 (23.8)
3 or more	8,292 (17.1)	11,209 (26.1)
**Smoking status**		
Non-smoker	17,415 (36.0)	20,602 (47.9)
Ex-smoker	15,188 (31.4)	9,916 (23.1)
Current smoker	9,359 (19.3)	7,448 (17.3)
Missing smoking status	6,451 (13.3)	5,034 (11.7)
**Index of Multiple Deprivation (IMD) quintile**		
1 – least deprived	11,490 (23.7)	9,229 (21.5)
2	10,850 (22.4)	9,275 (21.6)
3	8,858 (18.3)	7,896 (18.4)
4	7,859 (16.2)	7,413 (17.2)
5 – most deprived	6,310 (13.0)	6,304 (14.7)
Missing IMD	3,046 (6.3)	2,883 (6.7)

For peer review only

For peer review only - http://bmjopen.bmj.com/site/about/guidelines.xhtml

The majority of patients did not receive a weight management intervention during the study period. The proportion of patients by BMI category with each type of intervention recorded on their medical record is given in Table 2. In patients with morbid obesity, 60.0% of men and 58.1% of women had no record of weight management during the 7 years of the study. In patients with non-severe obesity (BMI 30-34.9kg/m^2) the figures were 84.2% and 80.2% respectively. The proportion of patients who received an intervention increased with each additional BMI category. Advice was the most commonly recorded intervention in overweight and obese patients and severely obese men. Drug interventions were the most frequently recorded intervention in morbidly obese patients and severely obese women.

Table 2: Patients who received, or did not receive, a weight management intervention over the study period by gender and BMI category. Figures are frequencies (row percent).

BMI category	Total	Advice	Referral	Drugs	No treatment
		**Men**			
Overweight (BMI 25-29.9)	30,950	1,805 (5.8)	913 (2.9)	86 (0.3)	28,282 (91.4)
Obese (BMI 30-34.9)	12,711	1,129 (8.9)	762 (6.0)	313 (2.5)	10,697 (84.2)
Severe obesity (BMI 35-39.9)	3,368	363 (10.8)	349 (10.4)	333 (9.9)	2,499 (74.2)
Morbid obesity (BMI ≥40)	1,384	168 (12.1)	239 (17.3)	322 (23.3)	831 (60.0)
		**Women**			
Overweight (BMI 25-29.9)	24,144	1,331 (5.5)	762 (3.2)	451 (1.9)	21,794 (90.3)
Obese (BMI 30-34.9)	11,364	925 (8.1)	740 (6.5)	889 (7.8)	9,116 (80.2)
Severe obesity (BMI 35-39.9)	4,777	462 (9.7)	445 (9.3)	671 (14.0)	3,460 (72.4)
Morbid obesity (BMI ≥40)	2,715	284 (10.5)	479 (17.6)	724 (26.7)	1,578 (58.1)

For peer review only - http://bmjopen.bmj.com/site/about/guidelines.xhtml

Rates of intervention are presented in Table 3. Overall, the recorded rates of intervention were highest for advice at 30.3 (95% CI 29.3 to 31.4) per 1,000 person-years. The rate of each intervention type increased in higher BMI categories. The rate of advice was 22.6 (21.6 to 23.8) per 1,000 in overweight patients, and highest at 60.2 (51.8 to 70.4) per 1,000 in morbidly obese patients. In overweight patients, advice was the most commonly used intervention, whereas drug prescription was the most common in morbidly obese patients.

Table 3: Rate of obesity management intervention by body mass index (BMI) category (per 1000 patient years), based on records of advice, referral or obesity drug prescription.

	Advice	Referral	Drugs
**Overall rate**	30.3 (29.3 to 31.4)	20.0 (19.3 to 20.8)	13.9 (13.5 to 14.4)
**Overweight (BMI 25-29.9)**	22.6 (21.6 to 23.8)	11.2 (10.5 to 11.9)	2.9 (2.6 to 3.2)
**Obese (BMI 30-34.9)**	36.4 (34.4 to 38.6)	23.7 (22.3 to 25.2)	15.7 (14.7 to 16.7)
**Severe obesity (BMI 35-39.9)**	47.2 (42.7 to 52.3)	38.4 (35.4 to 41.7)	41.5 (39.0 to 44.1)
**Morbid obesity (BMI ≥40)**	60.2 (51.8 to 70.4)	75.7 (69.5 to 82.6)	89.9 (85.0 to 95.2)

For peer review only - http://bmjopen.bmj.com/site/about/guidelines.xhtml

The multivariable analysis identified BMI category as the strongest predictor of weight-loss intervention, with a hazard ratio (HR) of 1.68 (95% CI 1.60 to 1.76) for obesity and 3.67 (95% CI 3.45 to 3.92) for morbid obesity (Table 4). Increasing age, type 2 diabetes and depression were also strong predictors of patients receiving a weight loss intervention. Female gender, being a former smoker and socioeconomic deprivation were associated with treatment for overweight and obesity.

Table 4: Cox proportional hazards model investigating time to multiple weight management interventions after a record of overweight or obesity.

	Patients receiving weight management intervention	Total patients	Hazard ratio	95% CI	P value
**Age (decades)**	-	-	1.42	1.27 to 1.58	<0.001
**Age squared**	-	-	0.97	0.96 to 0.98	<0.001
**Gender**					
Male	6,104	48,413	1.00	-	-
Female	7,054	43,000	1.14	1.10 to 1.19	<0.001
**BMI group***					
Overweight (BMI 25-29.9)	5,019	50,075	1.00	-	-
Obese (BMI 30-34.9)	4,263	19,812	1.68	1.60 to 1.76	<0.001
Severe obesity (BMI 35-39.9)	2,186	5,959	2.36	2.23 to 2.50	<0.001
Morbid obesity (BMI ≥40)	1,690	2,409	3.67	3.45 to 3.91	<0.001
**Smoking status**					
Non-smoker	5,441	32,576	1.00	-	-
Former smoker	3,962	24,142	1.11	1.06 to 1.16	<0.001
Current smoker	2,530	14,277	0.99	0.94 to 1.05	0.823
Missing smoking status	1,225	10,260	0.82	0.77 to 0.88	<0.001
**Index of Multiple Deprivation quintile**					
1 - least deprived	2,564	18,155	1.00	-	-
2	2,490	17,635	0.94	0.88 to 1.00	0.054
3	2,511	14,243	1.20	1.12 to 1.29	<0.001
4	2,413	12,859	1.13	1.06 to 1.21	<0.001
5 – most deprived	2,277	10,337	1.24	1.15 to 1.32	<0.001
Missing IMD	903	5,026	1.04	0.95 to 1.13	0.395
**CHD**	1,993	9,669	1.24	1.16 to 1.31	<0.001
**Stroke**	535	2,603	1.09	0.98 to 1.21	0.116
**Type 2 diabetes**	4,401	12,884	1.83	1.75 to 1.92	<0.001
**Depression**	6,385	31,573	1.33	1.28 to 1.39	<0.001

* BMI group at baseline. Patients could change BMI category in the analysis so intervention may have been delivered when patients had changed BMI category

For peer review only - http://bmjopen.bmj.com/site/about/guidelines.xhtml

For peer review only

There was substantial variation between practices in the recording of obesity management interventions (see Table 5). The median proportion of obese and overweight patients receiving a weight management intervention during the study was 12% (Interquartile range (IQR) 7 to 19). A maximum of 91% overweight or obese patients in a practice had an intervention recorded. Follow-up measurements of body weight after intervention were most frequent in patients who had a referral, with 34.1% of patients having a weight measurement in the first year. In contrast, 20.7% of patients had a follow-up weight measurement in the first year after advice and 24.3% after a drug prescription. No trend in weight change was observed in patients up to five years after any of the three intervention types investigated.

Table 5: Distribution of general practice-specific proportions of overweight and obese patients receiving weight management interventions over the study period, variability among 491 GP practices.

	Minimum	10th percentile	25th percentile	Median	75th percentile	90th percentile	Maximum
**Patients receiving any intervention (%)**	0	4	7	12	19	28	91
**Patients receiving advice (%)**	0	0	0	3	9	18	91
**Patients receiving a referral (%)**	0	0	1	3	7	13	50
**Patients receiving anti-obesity drugs (%)**	0	0	2	4	6	9	33

NB: different practices may occupy centiles for different measures

For peer review only - http://bmjopen.bmj.com/site/about/guidelines.xhtml

For peer review only

DISCUSSION

Summary

Analysis of primary care electronic health records reveals that the use of weight management interventions in primary care for the treatment of overweight and obesity were infrequent between 2005 and 2012. The likelihood of intervention was strongly associated with BMI category. However, 60% of men and 58% of women with morbid obesity did not have any record of receiving weight management in primary care, with higher proportions noted in lower BMI categories. Variation in obesity management between general practices was evident, with many practices not recording any intervention. These results might be a consequence of poor documentation of advice given, but might also indicate a lack of access to appropriate body weight management interventions in primary care.

There was some evidence body weight management was tailored to obesity category with more frequent utilisation of anti-obesity drugs in patients who were in higher obesity categories and advice used more commonly in overweight patients. BMI category was the strongest predictor of a patient receiving weight management interventions, with rates over 3-times higher in morbid obesity than in overweight. Female gender, increasing age, socioeconomic deprivation and co-morbidities were also associated with greater use of weight management interventions.

Follow-up values for body weight after a recorded weight management intervention were limited. Monitoring of body weight in primary care is generally opportunistic and depends on patients attending the practice and having a weight measurement recorded. However, the relatively high levels of co-morbidity in patients in this cohort, including those that

For peer review only - http://bmjopen.bmj.com/site/about/guidelines.xhtml

require long-term management such as type 2 diabetes, suggest that consultations are likely to be regular. While follow-up weight measurements did not show any change in weight after intervention; these results are very vulnerable to information bias.

Comparison with the literature

One other UK-based study investigated using of primary care interventions for the treatment of obesity.(9) The Counterweight report identified that 20% of patients received advice, 4% referrals and 2% anti-obesity drugs based on a review of 100 obese patients medical records over an 18-month period in 2000-01. We identified a smaller proportion of patients receiving advice and a higher proportion having a referral or drug prescription over a longer time period. It was not clear how obese participants were selected in the Counterweight study. Other differences between the present study and the Counterweight paper include a larger sample size and inclusion of overweight patients. However, the results suggest that prescribing of anti-obesity drugs has increased in the last 15 years and rates of recorded advice may have fallen. Increased use of anti-obesity drugs between 1998, when they were first introduced in the UK, and 2005 has been reported elsewhere.(16)

A decline in lifestyle advice and counselling for weight loss given to obese patients over the last 10 years has been also been reported in studies from the US. Reasons behind this reduction, despite increasing obesity levels, include poor recording of advice, lack of time in consultations, pessimism regarding potential success of weight loss attempts and increased use of medications to treat obesity-related risk factors and disease (17, 18) and, perhaps, normalisation of excessive body weight. Although the evidence from the current study is not sufficient to conclude that a reduction in advice for weight management has

For peer review only - http://bmjopen.bmj.com/site/about/guidelines.xhtml

For peer review only

occurred, some of the explanations attributed to lowered rates in the US are likely to be applicable in the UK.

Strengths and limitations

This study design had the advantage of a large population-based sample taken from different regions of the UK. However, it is likely that not all weight management interventions, particularly lifestyle advice, were captured in the electronic health record. Brief advice may be given to patients but not recorded by clinicians, which could have led to an underestimation of intervention rates. This is less likely to be an issue with referrals and drug prescribing. Furthermore, the patients included in this sample were selected on the basis of having a BMI record indicating that they were overweight or obese. This may have introduced a selection bias as these patients have been identified as having a weight problem by a clinician. Patients who are obese but do not have a record of weight status in their medical record may or may not be receiving weight management interventions differently from those who have been diagnosed.

Implications for practice and future research

The results of this study suggest that primary-care interventions given to patients with the aim of reducing weight are under-utilised, and that follow-up to determine their success is poor. It is possible that rates have been under-estimated through a lack of formal recording in medical records. However, the growing burden of obesity on primary healthcare services and lack of long-term follow-up on the effectiveness of these treatments supports the use of structured recording of interventions for weight management and subsequent follow-up. This is particularly true given mixed results from reviews of the effectiveness of primary-care interventions for obesity and the need for further evidence specific to patient

For peer review only - http://bmjopen.bmj.com/site/about/guidelines.xhtml

sub-groups e.g. those with co-morbidities (8, 19, 20). Primary care referrals to commercial weight loss programmes have been found to be effective in trials.(21) Although this type of referral wasn't included in the present study, an analysis using primary care data could be valuable. Consistency of public health messages on the health risks associated with obesity should be promoted in primary care where clinicians have the opportunity to reach a large number of patients and utilise preventive as well as reactive treatment strategies.

Study funding: This study is based in part on data from the Clinical Practice Research Datalink (CPRD) obtained under license from the UK Medicines and Healthcare products Regulatory Agency (UKMHRA). This research was supported by the National Institute for Health Research (NIHR) Biomedical Research Centre at Guy's and St Thomas' NHS Foundation Trust and King's College London, and by the UK National Prevention Research Initiative (UKNPRI). The views expressed are those of the authors alone.

Competing interests: None declared

Contributions:

HB & MG designed the study. TP & MG advised on the conduct of the data analysis. HB conducted the analysis and drafted the paper. MG & TP contributed to interpretation of the results. All authors commented and approved the manuscript. HB is guarantor.

For peer review only - http://bmjopen.bmj.com/site/about/guidelines.xhtml

REFERENCES

1. Lopez AD, Mathers CD, Ezzati M, editors. Global burden of disease and risk factors. Washington: The World Bank & Oxford University Press; 2006.
2. Moody A. Chapter 10: Adult anthropometric measures, overweight and obesity. London, UK: Health and Social Care Information Centre, 2013.
3. National Institute for Health and Care Excellence. Obesity: Identification, assessment and management of overweight and obesity in children, young people and adults (draft for consultation). London, UK: National Institute for Health and Care Excellence, 2014 Contract No.: CG43.
4. Ara R, Blake L, Gray L, et al. What is the clinical effectiveness and cost-effectiveness of using drugs in treating obese patients in primary care? A systematic review. Health Technology Assessment. 2012;16(5).
5. Avenell A, Broom J, Brown TJ, et al. Systematic review of the long-term effects and economic consequences of treatments for obesity and implications for health improvement. Health Technology Assessment. 2004;8(21).
6. Picot J, Jones J, Colquitt J, et al. Weight Loss Surgery for Mild to Moderate Obesity: A Systematic Review and Economic Evaluation. Obesity Surgery. 2012;22(9):1496-506.
7. Picot J, Jones J, Colquitt JL, et al. The clinical effectiveness and cost-effectiveness of bariatric (weight loss) surgery for obesity: a systematic review and economic evaluation. Health Technology Assessment. 2009;13(41):215-357.
8. Booth HP, Prevost AT, Wright AJ, et al. (in press) Effectiveness of behavioral weight loss interventions delivered in a primary care setting: A systematic review and meta-analysis. Family Practice. 2014.
9. Counterweight Project Team. Current approaches to obesity management in UK primary care: the Counterweight programme. J Hum Nutr Diet. 2004;17:7.

For peer review only

For peer review only - http://bmjopen.bmj.com/site/about/guidelines.xhtml

10. Department of Health. Healthy Lives, Healthy People: a call to action on obesity. London: HM Government, 2011 Contract No.: 16166.

11. Williams T, van Staa T, Puri S, et al. Recent advances in the utility and use of the General Practice Research Database as an example of a UK Primary Care Data resource. Therapeutic Advances in Drug Safety. 2012;3(2):89-99.

12. Medicines and Healthcare Products Regulatory Agency. Suspension of sibutramine (Reductil) 2010 [19/03/2014]. Available from: http://www.mhra.gov.uk/NewsCentre/CON068470.

13. Medicines and Healthcare Products Regulatory Agency. Rimonabant: European suspension of marketing authorisation 2008 [cited 26/08/2014]. Available from: http://www.mhra.gov.uk/Safetyinformation/DrugSafetyUpdate/CON087927.

14. Kang JG, Park C-Y. Anti-Obesity Drugs: A Review about Their Effects and Safety. Diabetes Metab J. 2012;36(1):13–25.

15. Cleves M, StataCorp. Analysis of multiple failure-time data with Stata 2009 [cited 10/09/2014]. Available from: http://www.stata.com/support/faqs/statistics/multiple-failure-time-data/.

16. Srishanmuganathan J, Patel H, Car J, Majeed A. National trends in the use and costs of anti-obesity medications in England 1998–2005. J Public Health. 2007;29(2):199-202.

17. Yates EA, Macpherson AK, Kuk JL. Secular Trends in the Diagnosis and Treatment of Obesity Among US Adults in the Primary Care Setting. Obesity. 2012;20(9):1909-14.

18. McAlpine DDW, A R. Trends in Obesity-Related Counseling in Primary Care: 1995–2004. Medical Care. 2007;45(4):322-9.

19. Tsai AG, Wadden TA. Treatment of obesity in primary care practice in the United States: A systematic review. J Gen Intern Med. 2009;24(9):1073-9.

For peer review only - http://bmjopen.bmj.com/site/about/guidelines.xhtml

20. LeBlanc ES, O'Connor E, Whitlock EP, et al. Effectiveness of Primary Care–Relevant Treatments for Obesity in Adults: A Systematic Evidence Review for the U.S. Preventive Services Task Force. Ann Int Med. 2011;155(7):434-47.

21. Jebb SA, Ahern AL, Olson AD, et al. Primary care referral to a commercial provider for weight loss treatment versus standard care: a randomised controlled trial. The Lancet. 2011;378(9801):1485-92.

For peer review only

For peer review only - http://bmjopen.bmj.com/site/about/guidelines.xhtml

STROBE Statement—checklist of items that should be included in reports of observational studies

	Item No	Recommendation
**Title and abstract**	1	(*a*) Indicate the study's design with a commonly used term in the title or the abstract [p1]
		(*b*) Provide in the abstract an informative and balanced summary of what was done and what was found [p2]
**Introduction**		
Background/rationale	2	Explain the scientific background and rationale for the investigation being reported [p4, 2nd para]
Objectives	3	State specific objectives, including any prespecified hypotheses [abstract, p2 and p5]
**Methods**		
Study design	4	Present key elements of study design early in the paper [p6]
Setting	5	Describe the setting, locations, and relevant dates, including periods of recruitment, exposure, follow-up, and data collection [p6]
Participants	6	(*a*) *Cohort study*—Give the eligibility criteria, and the sources and methods of selection of participants. Describe methods of follow-up [p6]<br>*Case-control study*—Give the eligibility criteria, and the sources and methods of case ascertainment and control selection. Give the rationale for the choice of cases and controls<br>*Cross-sectional study*—Give the eligibility criteria, and the sources and methods of selection of participants
		(*b*) *Cohort study*—For matched studies, give matching criteria and number of exposed and unexposed<br>*Case-control study*—For matched studies, give matching criteria and the number of controls per case
Variables	7	Clearly define all outcomes, exposures, predictors, potential confounders, and effect modifiers. Give diagnostic criteria, if applicable [p6 – exposure and outcome definitions]
Data sources/ measurement	8*	For each variable of interest, give sources of data and details of methods of assessment (measurement). Describe comparability of assessment methods if there is more than one group [p6 –7]
Bias	9	Describe any efforts to address potential sources of bias
Study size	10	Explain how the study size was arrived at
Quantitative variables	11	Explain how quantitative variables were handled in the analyses. If applicable, describe which groupings were chosen and why [p6 – exposure and outcome definitions]
Statistical methods	12	(*a*) Describe all statistical methods, including those used to control for confounding [p7-8]
		(*b*) Describe any methods used to examine subgroups and interactions
		(*c*) Explain how missing data were addressed
		(*d*) *Cohort study*—If applicable, explain how loss to follow-up was addressed<br>*Case-control study*—If applicable, explain how matching of cases and controls was addressed<br>*Cross-sectional study*—If applicable, describe analytical methods taking account of

For peer review only - http://bmjopen.bmj.com/site/about/guidelines.xhtml

	sampling strategy
	(*e*) Describe any sensitivity analyses

Continued on next page

For peer review only

**Results**		
Participants	13*	(a) Report numbers of individuals at each stage of study—eg numbers potentially eligible, examined for eligibility, confirmed eligible, included in the study, completing follow-up, and analysed
		(b) Give reasons for non-participation at each stage
		(c) Consider use of a flow diagram
Descriptive data	14*	(a) Give characteristics of study participants (eg demographic, clinical, social) and information on exposures and potential confounders [p9 & Table 1]
		(b) Indicate number of participants with missing data for each variable of interest [Table 1]
		(c) *Cohort study*—Summarise follow-up time (eg, average and total amount)
Outcome data	15*	*Cohort study*—Report numbers of outcome events or summary measures over time [p11-12]
		Case-control study—Report numbers in each exposure category, or summary measures of exposure
		Cross-sectional study—Report numbers of outcome events or summary measures
Main results	16	(*a*) Give unadjusted estimates and, if applicable, confounder-adjusted estimates and their precision (eg, 95% confidence interval). Make clear which confounders were adjusted for and why they were included [throughout results]
		(*b*) Report category boundaries when continuous variables were categorized [p6 – BMI]
		(*c*) If relevant, consider translating estimates of relative risk into absolute risk for a meaningful time period
Other analyses	17	Report other analyses done—eg analyses of subgroups and interactions, and sensitivity analyses
**Discussion**		
Key results	18	Summarise key results with reference to study objectives [p15]
Limitations	19	Discuss limitations of the study, taking into account sources of potential bias or imprecision. Discuss both direction and magnitude of any potential bias [p17]
Interpretation	20	Give a cautious overall interpretation of results considering objectives, limitations, multiplicity of analyses, results from similar studies, and other relevant evidence [p17]
Generalisability	21	Discuss the generalisability (external validity) of the study results [p17]
**Other information**		
Funding	22	Give the source of funding and the role of the funders for the present study and, if applicable, for the original study on which the present article is based [p18]

*Give information separately for cases and controls in case-control studies and, if applicable, for exposed and unexposed groups in cohort and cross-sectional studies.

Note: An Explanation and Elaboration article discusses each checklist item and gives methodological background and published examples of transparent reporting. The STROBE checklist is best used in conjunction with this article (freely available on the Web sites of PLoS Medicine at http://www.plosmedicine.org/, Annals of Internal Medicine at http://www.annals.org/, and Epidemiology at http://www.epidem.com/). Information on the STROBE Initiative is available at www.strobe-statement.org.

For peer review only

BMJ Open

Access to weight reduction interventions for overweight and obese patients in UK primary care. Population-based cohort study

Journal:	*BMJ Open*
Manuscript ID:	bmjopen-2014-006642.R1
Article Type:	Research
Date Submitted by the Author:	16-Oct-2014
Complete List of Authors:	Booth, Helen; King's College London, Primary Care and Public Health Sciences<br>Prevost, A.; King's College London, Primary Care and Public Health Sciences<br>Gulliford, Martin; King's College London, Primary Care and Public Health Sciences
**Primary Subject Heading**:	General practice / Family practice
Secondary Subject Heading:	Public health
Keywords:	PRIMARY CARE, PUBLIC HEALTH, EPIDEMIOLOGY

SCHOLARONE™ Manuscripts

For peer review only

Access to weight reduction interventions for overweight and obese patients in UK primary care. Population-based cohort study

Running head: Obesity management in primary care

Article category: Original research

Authors:

Helen P. Booth[1], BSc MSc

A. Toby Prevost[1], BSc MSc PhD

Martin C. Gulliford[1], FFPH FRCP

[1]Department of Primary Care and Public Health Sciences, King's College London, UK

Corresponding author:

Miss Helen P. Booth, Research Associate, Department of Primary Care and Public Health Sciences, King's College London, 7th Floor Capital House, 42 Weston Street, London,

SE1 3QD. Tel: 020 7848 6104. Fax: 020 7848 6620 Email: helen.booth@kcl.ac.uk

References: 21

Tables: 5

Figures: 0

Word count: 3,403

Abstract word count: 271

Supplementary files: 0

Key words: Obesity, Overweight, Primary health care, General practice

For peer review only

ABSTRACT

Objectives

To investigate access to weight management interventions for overweight and obese patients in primary care

Setting

UK primary care electronic health records

Participants

A cohort of 91,413 overweight and obese patients aged 30 to 100 years was sampled from the Clinical Practice Research Datalink (CPRD). Patients with body mass index (BMI) values ≥25 kg/m^2 recorded between 2005 and 2012 were included. BMI values were categorised using World Health Organization (WHO) criteria.

Interventions

Interventions for body weight management, including advice, referrals and prescription of anti-obesity drugs, were evaluated.

Primary and secondary outcome measures

The rate of body weight management interventions and time to intervention were the main outcomes

Results

Data were analysed for 91,413 patients, mean age 56 years, including 55,094 (60%) overweight and 36,319 (40%) obese, including 4,099 (5%) with morbid obesity. During the study period 90% of overweight patients had no weight management intervention recorded. Intervention was more frequent among obese patients, but 59% of patients with morbid obesity had no intervention recorded. Rates of

For peer review only

intervention increased with BMI category. In morbid obesity, rates of intervention per 1,000 patient years were: advice, 60.2 (95% CI 51.8 to 70.4); referral, 75.7 (95% CI 69.5 to 82.6); and anti-obesity drugs 89.9 (95% CI 85.0 to 95.2). Weight management interventions were more often accessed by women, older patients, those with co-morbidity and those in deprivation. Follow-up of body weight subsequent to interventions was infrequent.

Conclusions

Limited evidence of weight management interventions in primary care electronic health records may result from poor recording of advice given, but may indicate a lack of patient access to appropriate body weight management interventions in primary care.

ARTICLE SUMMARY

Strengths and limitations of this study

- This study uses primary care electronic health records to investigate the use of weight management interventions in overweight and obese patients
- Lifestyle advice was the most commonly-used intervention in all but morbidly obese patients, where anti-obesity drugs were more frequent
- Patients had to have a BMI value recorded to be included in the study
- Clinicians may be giving advice for weight management but not recording it

For peer review only

BACKGROUND

Obesity is a leading cause of premature morbidity and mortality worldwide.(1) In the UK, a quarter of adults are obese and up to two thirds are overweight.(2) Primary care represents an important setting in which obese patients may access weight loss interventions. The main strategies for treating obesity are provision of lifestyle advice, referral for weight management, prescription of anti-obesity drugs and, in severe cases, referral for bariatric surgery. Clinical guidelines recommend a stepped approach to weight management depending on the severity of a patient's obesity and whether they have weight-related co-morbidities, with more intensive interventions offered as appropriate. Interventions should be agreed between the clinician and patient, and offered in conjunction with long-term follow-up and continuing care.(3)

Several studies have evaluated the effectiveness of primary care interventions for weight management in obesity, (4-8) but few studies have evaluated how overweight and obese patients are managed in primary care. A survey using data collected in 2000/1, before the UK national guidelines on obesity management were published,(9) found that a fifth of obese patients were offered dietary counselling, less than 5% a referral and 2% anti-obesity medications over an 18 month period. There is no more recent information on the use of interventions for the management of obesity in primary care.

Access to appropriate weight management interventions for overweight and obese patients in primary care is of increasing importance in the context of a national objective to establish a downward trend in obesity among UK adults by 2020.(10)

For peer review only - http://bmjopen.bmj.com/site/about/guidelines.xhtml

This study aimed to evaluate access in terms of recording and utilisation of weight management interventions for overweight and obesity using primary care electronic health records. Interventions were classified as lifestyle advice, referrals for weight management and prescription of anti-obesity drugs.

For peer review only

For peer review only - http://bmjopen.bmj.com/site/about/guidelines.xhtml

METHODS

Data source and cohort definition

A cohort of patients was selected from the Clinical Practice Research Datalink (CPRD), a database of longitudinal patient electronic medical records from UK primary care. CPRD is the largest primary care database in the world, and represents over 5% of the UK population with about 680 practices currently contributing research quality data.(11) The initial cohort was selected as part of a larger project, and comprised a random sample of approximately 300,000 patients who were registered for at least 12 months with a general practice contributing data to CPRD between the 1st January 2005 and 30th April 2012. Equal numbers of men and women were drawn from each year of the study without replacement. Patients were selected who had a body mass index (BMI) record indicating overweight or obesity during the study period. The study was approved by the CPRD Independent Scientific Advisory Committee (ISAC 07_054 and 14_056).

Exposure and outcome definitions

BMI was categorised using the World Health Organization (WHO) categories: overweight (BMI 25-29.9kg/m^2), obese (BMI 30-34.9kg/m^2), severe obesity (BMI 35.0 to 39.9kg/m^2), morbid obesity (BMI≥40kg/m^2). Medical diagnoses of obesity in the medical record were also noted based on the presence of diagnostic codes. Morbidity status was ascertained based on the presence of eleven common conditions associated with obesity, including: type 2 diabetes, coronary heart disease (CHD), stroke, depression, osteoarthritis, back pain, joint problems, cancer, gallbladder disease, asthma and sleep apnoea. Smoking status and socioeconomic

For peer review only - http://bmjopen.bmj.com/site/about/guidelines.xhtml

deprivation were also included as exposures. Socioeconomic deprivation was classified into quintiles using the Index of Multiple Deprivation rank based on patient postcode. Data on deprivation were only available for patients registered at English practices.

Interventions for the management of body weight were identified using medical codes recorded in clinical and referral records, recorded health promotion advice, and prescriptions for anti-obesity drugs. For analysis, weight management interventions were classified into lifestyle advice, referrals for weight management and prescription of anti-obesity drugs. Advice included codes relating to dieting, exercise and weight loss. Relevant referrals included those to community and hospital dieticians, for exercise therapy and for weight management programmes. Prescriptions for three different anti-obesity drugs were included; two of these, sibutramine and rimonabant, have been removed from the UK market because of safety concerns.(12, 13) However, these drugs were in use during the time period investigated and so have been considered in this analysis. The only anti-obesity drug currently licensed in the UK, orlistat, was introduced in 1998 and has been available over the counter as well as by prescription since 2009.(14) Multiple prescriptions of anti-obesity drugs were considered to be a part of the same course of treatment if there was less than 6 months between prescriptions.

Analysis

Person time was analysed following the index date; the first BMI record for overweight or obesity after the 1st January 2005. Patient baseline characteristics were tabulated. The proportion of patients who received weight management

For peer review only - http://bmjopen.bmj.com/site/about/guidelines.xhtml

interventions over the study period was evaluated by BMI category. Time-to-event analysis was used to calculate the rate of intervention utilisation by BMI category and to investigate variables associated with intervention using a multiple-failure multivariable Cox proportional hazards regression model with unordered events.(15) Variation in the use of weight management interventions by GP practice was investigated by calculating the proportion of patients receiving any intervention in the year following the index date. These data were then presented as percentiles of the distribution for all practices. Change in weight from baseline after the implementation of each type of intervention was calculated for up to 5 years of follow-up.

For peer review only - http://bmjopen.bmj.com/site/about/guidelines.xhtml

RESULTS

Of the 300,006 patients in the cohort, 134,697 (45%) had an eligible BMI record. After patients with BMIs lower than $25kg/m^2$ were removed, data were analysed for 91,413 patients, with mean age 56 years, including 55,094 (60%) overweight and 36,319 (40%).or obese, including 4,099 (5%) with morbid obesity. Mean age in men and women was 56 years. Patient characteristics on entry to the study are presented in Table 1. At the index date (date of the first relevant BMI record) most patients were overweight (63.9% of men and 56.2% of women); 2.9% of men and 6.3% of women were morbidly obese. A diagnostic code for obesity was recorded for 3.9% of male patients and 6.5% of females. A higher proportion of women were non-smokers, while men were more likely to be former or current smokers.

For peer review only

Table 1: Characteristics of overweight and obese patients. Figures are frequencies (column percent) unless stated otherwise.

	Men (48,413)	Women (43,000)
**Mean age (SD)**	55.6 (13.9)	56.4 (15.0)
**Body mass index (BMI) category (Kg/m2)**		
Overweight (BMI 25-29.9)	30,950 (63.9)	24,144 (56.2)
Obese (BMI 30-34.9)	12,711 (26.3)	11,364 (26.4)
Severe obesity (BMI 35-39.9)	3,368 (7.0)	4,777 (11.1)
Morbid obesity (BMI ≥40)	1,384 (2.9)	2,715 (6.3)
**Medical code for obesity**	1,876 (3.9)	2,810 (6.5)
**Number of morbidities**		
0	14,810 (30.6)	9,635 (22.4)
1	14,988 (31.0)	11,919 (27.7)
2	10,323 (21.3)	10,237 (23.8)
3 or more	8,292 (17.1)	11,209 (26.1)
**Smoking status**		
Non-smoker	17,415 (36.0)	20,602 (47.9)
Ex-smoker	15,188 (31.4)	9,916 (23.1)
Current smoker	9,359 (19.3)	7,448 (17.3)
Missing smoking status	6,451 (13.3)	5,034 (11.7)
**Index of Multiple Deprivation (IMD) quintile**		
1 – least deprived	11,490 (23.7)	9,229 (21.5)
2	10,850 (22.4)	9,275 (21.6)
3	8,858 (18.3)	7,896 (18.4)
4	7,859 (16.2)	7,413 (17.2)
5 – most deprived	6,310 (13.0)	6,304 (14.7)
Missing IMD	3,046 (6.3)	2,883 (6.7)

For peer review only - http://bmjopen.bmj.com/site/about/guidelines.xhtml

The majority of patients did not receive a weight management intervention during the study period. The proportion of patients by BMI category with each type of intervention recorded on their medical record is given in Table 2. In patients with morbid obesity, 60.0% of men and 58.1% of women had no record of weight management during the 7 years of the study. In patients with non-severe obesity (BMI 30-34.9kg/m^2) the figures were 84.2% and 80.2% respectively. The proportion of patients who received an intervention increased with each additional BMI category. Advice was the most commonly recorded intervention in overweight and obese patients and severely obese men. Drug interventions were the most frequently recorded intervention in morbidly obese patients and severely obese women.

Table 2: Patients who received, or did not receive, a weight management intervention over the study period by gender and BMI category. Figures are frequencies (row percent).

BMI category	Total	Advice	Referral	Drugs	No treatment
		**Men**			
Overweight (BMI 25-29.9)	30,950	1,805 (5.8)	913 (2.9)	86 (0.3)	28,282 (91.4)
Obese (BMI 30-34.9)	12,711	1,129 (8.9)	762 (6.0)	313 (2.5)	10,697 (84.2)
Severe obesity (BMI 35-39.9)	3,368	363 (10.8)	349 (10.4)	333 (9.9)	2,499 (74.2)
Morbid obesity (BMI ≥40)	1,384	168 (12.1)	239 (17.3)	322 (23.3)	831 (60.0)
		**Women**			
Overweight (BMI 25-29.9)	24,144	1,331 (5.5)	762 (3.2)	451 (1.9)	21,794 (90.3)
Obese (BMI 30-34.9)	11,364	925 (8.1)	740 (6.5)	889 (7.8)	9,116 (80.2)
Severe obesity (BMI 35-39.9)	4,777	462 (9.7)	445 (9.3)	671 (14.0)	3,460 (72.4)
Morbid obesity (BMI ≥40)	2,715	284 (10.5)	479 (17.6)	724 (26.7)	1,578 (58.1)

For peer review only - http://bmjopen.bmj.com/site/about/guidelines.xhtml

Rates of intervention are presented in Table 3. Overall, the recorded rates of intervention were highest for advice at 30.3 (95% CI 29.3 to 31.4) per 1,000 person-years. The rate of each intervention type increased in higher BMI categories. The rate of advice was 22.6 (21.6 to 23.8) per 1,000 in overweight patients, and highest at 60.2 (51.8 to 70.4) per 1,000 in morbidly obese patients. In overweight patients, advice was the most commonly used intervention, whereas drug prescription was the most common in morbidly obese patients.

Table 3: Rate of obesity management intervention by body mass index (BMI) category (per 1000 patient years), based on records of advice, referral or obesity drug prescription.

	Advice	Referral	Drugs
**Overall rate**	30.3 (29.3 to 31.4)	20.0 (19.3 to 20.8)	13.9 (13.5 to 14.4)
**Overweight (BMI 25-29.9)**	22.6 (21.6 to 23.8)	11.2 (10.5 to 11.9)	2.9 (2.6 to 3.2)
**Obese (BMI 30-34.9)**	36.4 (34.4 to 38.6)	23.7 (22.3 to 25.2)	15.7 (14.7 to 16.7)
**Severe obesity (BMI 35-39.9)**	47.2 (42.7 to 52.3)	38.4 (35.4 to 41.7)	41.5 (39.0 to 44.1)
**Morbid obesity (BMI ≥40)**	60.2 (51.8 to 70.4)	75.7 (69.5 to 82.6)	89.9 (85.0 to 95.2)

For peer review only - http://bmjopen.bmj.com/site/about/guidelines.xhtml

The multivariable analysis identified BMI category as the strongest predictor of weight-loss intervention, with a hazard ratio (HR) of 1.68 (95% CI 1.60 to 1.76) for obesity and 3.67 (95% CI 3.45 to 3.92) for morbid obesity (Table 4). Increasing age, type 2 diabetes and depression were also strong predictors of patients receiving a weight loss intervention. Female gender, being a former smoker and socioeconomic deprivation were associated with treatment for overweight and obesity.

Table 4: Cox proportional hazards model investigating time to multiple weight management interventions after a record of overweight or obesity.

	Patients receiving weight management intervention (n)	Total patients (N)	Hazard ratio	95% confidence interval	P value
**Age (decades)**	-	-	1.42	1.27 to 1.58	<0.001
**Age squared**	-	-	0.97	0.96 to 0.98	<0.001
**Gender**					
Male	6,104	48,413	1.00	-	-
Female	7,054	43,000	1.14	1.10 to 1.19	<0.001
**Body mass index (BMI) category***					
Overweight (BMI 25-29.9)	5,019	50,075	1.00	-	-
Obese (BMI 30-34.9)	4,263	19,812	1.68	1.60 to 1.76	<0.001
Severe obesity (BMI 35-39.9)	2,186	5,959	2.36	2.23 to 2.50	<0.001
Morbid obesity (BMI ≥40)	1,690	2,409	3.67	3.45 to 3.91	<0.001
**Smoking status**					
Non-smoker	5,441	32,576	1.00	-	-
Former smoker	3,962	24,142	1.11	1.06 to 1.16	<0.001
Current smoker	2,530	14,277	0.99	0.94 to 1.05	0.823
Missing smoking status	1,225	10,260	0.82	0.77 to 0.88	<0.001
**Index of Multiple Deprivation quintile**					
1 - least deprived	2,564	18,155	1.00	-	-
2	2,490	17,635	0.94	0.88 to 1.00	0.054
3	2,511	14,243	1.20	1.12 to 1.29	<0.001
4	2,413	12,859	1.13	1.06 to 1.21	<0.001
5 – most deprived	2,277	10,337	1.24	1.15 to 1.32	<0.001
Missing IMD	903	5,026	1.04	0.95 to 1.13	0.395
**Coronary heart disease (CHD)**	1,993	9,669	1.24	1.16 to 1.31	<0.001
**Stroke**	535	2,603	1.09	0.98 to 1.21	0.116
**Type 2 diabetes**	4,401	12,884	1.83	1.75 to 1.92	<0.001
**Depression**	6,385	31,573	1.33	1.28 to 1.39	<0.001

* BMI group at baseline. Patients could change BMI category in the analysis so intervention may have been delivered when patients had changed BMI category

For peer review only - http://bmjopen.bmj.com/site/about/guidelines.xhtml

There was substantial variation between practices in the recording of obesity management interventions (see Table 5). The median proportion of obese and overweight patients receiving a weight management intervention during the study was 12% (Interquartile range (IQR) 7 to 19). A maximum of 91% overweight or obese patients in a practice had an intervention recorded. Follow-up measurements of body weight after intervention were most frequent in patients who had a referral, with 34.1% of patients having a weight measurement in the first year. In contrast, 20.7% of patients had a follow-up weight measurement in the first year after advice and 24.3% after a drug prescription. No trend in weight change was observed in patients up to five years after any of the three intervention types investigated.

Table 5: Use of weight management interventions in general practices. Figures are percentiles among 491 GP practices representing the proportion of patients in the practice receiving interventions.

	Minimum	10th percentile	25th percentile	Median	75th percentile	90th percentile	Maximum
**Patients receiving any intervention (%)**	0	4	7	12	19	28	91
**Patients receiving advice (%)**	0	0	0	3	9	18	91
**Patients receiving a referral (%)**	0	0	1	3	7	13	50
**Patients receiving anti-obesity drugs (%)**	0	0	2	4	6	9	33

NB: different practices may occupy centiles for different measures

For peer review only - http://bmjopen.bmj.com/site/about/guidelines.xhtml

DISCUSSION

Summary

Analysis of primary care electronic health records reveals that the use of weight management interventions in primary care for the treatment of overweight and obesity were infrequent between 2005 and 2012. The likelihood of intervention was strongly associated with BMI category. However, 60% of men and 58% of women with morbid obesity did not have any record of receiving weight management in primary care, with higher proportions noted in lower BMI categories. Variation in obesity management between general practices was evident, with many practices not recording any intervention. These results might be a consequence of poor documentation of advice given, but might also indicate a lack of patient access to appropriate body weight management interventions in primary care due to a lack of clinician awareness or confidence in treating obesity.

There was some evidence body weight management was tailored to obesity category with more frequent utilisation of anti-obesity drugs in patients who were in higher obesity categories and advice used more commonly in overweight patients. BMI category was the strongest predictor of a patient receiving weight management interventions, with rates over 3-times higher in morbid obesity than in overweight. Female gender, increasing age, socioeconomic deprivation and co-morbidities were also associated with greater use of weight management interventions.

Follow-up values for body weight after a recorded weight management intervention were limited. Monitoring of body weight in primary care is generally opportunistic and depends on patients attending the practice and having a weight measurement recorded. However,

For peer review only

the relatively high levels of co-morbidity in patients in this cohort, including those that require long-term management such as type 2 diabetes, suggest that consultations are likely to be regular. While follow-up weight measurements did not show any change in weight after intervention; these results are very vulnerable to information bias.

Comparison with the literature

One other UK-based study investigated using of primary care interventions for the treatment of obesity.(9) The Counterweight report identified that 20% of patients received advice, 4% referrals and 2% anti-obesity drugs based on a review of 100 obese patients medical records over an 18-month period in 2000-01. We identified a smaller proportion of patients receiving advice and a higher proportion having a referral or drug prescription over a longer time period. It was not clear how obese participants were selected in the Counterweight study. Other differences between the present study and the Counterweight paper include a larger sample size and inclusion of overweight patients. However, the results suggest that prescribing of anti-obesity drugs has increased in the last 15 years. Increased use of anti-obesity drugs between 1998, when they were first introduced in the UK, and 2005 has been reported elsewhere.(16)

A decline in lifestyle advice and counselling for weight loss given to obese patients over the last 10 years has been also been reported in studies from the US. Reasons behind this reduction, despite increasing obesity levels, include poor recording of advice, lack of time in consultations, pessimism regarding potential success of weight loss attempts and increased use of medications to treat obesity-related risk factors and disease (17, 18) and, perhaps, normalisation of excessive body weight. Although the evidence from the current study is not sufficient to conclude that a reduction in advice for weight management has

For peer review only

For peer review only - http://bmjopen.bmj.com/site/about/guidelines.xhtml

occurred, some of the explanations attributed to lowered rates in the US are likely to be applicable in the UK.

Strengths and limitations

This study design had the advantage of a large population-based sample taken from different regions of the UK. However, it is likely that not all weight management interventions, particularly lifestyle advice, were captured in the electronic health record. Brief advice may be given to patients but not recorded by clinicians, which could have led to an underestimation of intervention rates. This is less likely to be an issue with referrals and drug prescribing. Furthermore, the patients included in this sample were selected on the basis of having a BMI record indicating that they were overweight or obese. This may have introduced a selection bias as these patients have been identified as having a weight problem by a clinician. Patients who are obese but do not have a record of weight status in their medical record may or may not be receiving weight management interventions differently from those who have been diagnosed.

Implications for practice and future research

The results of this study suggest that primary-care interventions given to patients with the aim of reducing weight are under-utilised, and that follow-up to determine their success is poor. It is possible that rates have been under-estimated through a lack of formal recording in medical records. However, the growing burden of obesity on primary healthcare services and lack of long-term follow-up on the effectiveness of these treatments supports the use of structured recording of interventions for weight management and subsequent follow-up. This is particularly true given the heterogeneity of results from weight loss studies included in reviews of the effectiveness of primary-care interventions for obesity

For peer review only - http://bmjopen.bmj.com/site/about/guidelines.xhtml

and the need for further evidence specific to patient sub-groups e.g. those with co-morbidities (8, 19, 20). Primary care referrals to commercial weight loss programmes have been found to be effective in trials.(21-22) Although this type of referral wasn't included in the present study, an analysis using primary care data could be valuable. Data in CPRD are not specific enough to permit this at present. Consistency of public health messages on the health risks associated with obesity should be promoted in primary care where clinicians have the opportunity to reach a large number of patients and utilise preventive as well as reactive treatment strategies.

Study funding: This study was supported by the UK National Prevention Research Initiative whose funding partners include the Alzheimer's Research Trust; Alzheimer's Society; Biotechnology and Biological Sciences Research Council; British Heart Foundation; Cancer Research UK; Chief Scientist Office, Scottish Government Health Directorate; Department of Health; Diabetes UK; Economic and Social Research Council; Engineering and Physical Sciences Research Council; Health & Social Care Research & Development Office for Northern Ireland; Medical Research Council; The Stroke Association; Welsh Assembly Government; and World Cancer Research Fund.The views expressed are those of the authors alone.

Competing interests: None declared

Contributions: HB & MG designed the study. TP & MG advised on the conduct of the data analysis. HB conducted the analysis and drafted the paper. MG & TP contributed to interpretation of the results. All authors commented and approved the manuscript. HB is guarantor.

For peer review only - http://bmjopen.bmj.com/site/about/guidelines.xhtml

REFERENCES

1. Lopez AD, Mathers CD, Ezzati M, editors. Global burden of disease and risk factors. Washington: The World Bank & Oxford University Press; 2006.

2. Moody A. Chapter 10: Adult anthropometric measures, overweight and obesity. London, UK: Health and Social Care Information Centre, 2013.

3. National Institute for Health and Care Excellence. Obesity: Identification, assessment and management of overweight and obesity in children, young people and adults (draft for consultation). London, UK: National Institute for Health and Care Excellence, 2014 Contract No.: CG43.

4. Ara R, Blake L, Gray L, et al. What is the clinical effectiveness and cost-effectiveness of using drugs in treating obese patients in primary care? A systematic review. Health Technology Assessment. 2012;16(5).

5. Avenell A, Broom J, Brown TJ, et al. Systematic review of the long-term effects and economic consequences of treatments for obesity and implications for health improvement. Health Technology Assessment. 2004;8(21).

6. Picot J, Jones J, Colquitt J, et al. Weight Loss Surgery for Mild to Moderate Obesity: A Systematic Review and Economic Evaluation. Obesity Surgery. 2012;22(9):1496-506.

7. Picot J, Jones J, Colquitt JL, et al. The clinical effectiveness and cost-effectiveness of bariatric (weight loss) surgery for obesity: a systematic review and economic evaluation. Health Technology Assessment. 2009;13(41):215-357.

8. Booth HP, Prevost AT, Wright AJ, et al. (in press) Effectiveness of behavioral weight loss interventions delivered in a primary care setting: A systematic review and meta-analysis. Family Practice. 2014.

9. Counterweight Project Team. Current approaches to obesity management in UK primary care: the Counterweight programme. J Hum Nutr Diet. 2004;17:7.

For peer review only - http://bmjopen.bmj.com/site/about/guidelines.xhtml

For peer review only

10. Department of Health. Healthy Lives, Healthy People: a call to action on obesity. London: HM Government, 2011 Contract No.: 16166.

11. Williams T, van Staa T, Puri S, et al. Recent advances in the utility and use of the General Practice Research Database as an example of a UK Primary Care Data resource. Therapeutic Advances in Drug Safety. 2012;3(2):89-99.

12. Medicines and Healthcare Products Regulatory Agency. Suspension of sibutramine (Reductil) 2010 [19/03/2014]. Available from: http://www.mhra.gov.uk/NewsCentre/CON068470.

13. Medicines and Healthcare Products Regulatory Agency. Rimonabant: European suspension of marketing authorisation 2008 [cited 26/08/2014]. Available from: http://www.mhra.gov.uk/Safetyinformation/DrugSafetyUpdate/CON087927.

14. Kang JG, Park C-Y. Anti-Obesity Drugs: A Review about Their Effects and Safety. Diabetes Metab J. 2012;36(1):13–25.

15. Cleves M, StataCorp. Analysis of multiple failure-time data with Stata 2009 [cited 10/09/2014]. Available from: http://www.stata.com/support/faqs/statistics/multiple-failure-time-data/.

16. Srishanmuganathan J, Patel H, Car J, Majeed A. National trends in the use and costs of anti-obesity medications in England 1998–2005. J Public Health. 2007;29(2):199-202.

17. Yates EA, Macpherson AK, Kuk JL. Secular Trends in the Diagnosis and Treatment of Obesity Among US Adults in the Primary Care Setting. Obesity. 2012;20(9):1909-14.

18. McAlpine DDW, A R. Trends in Obesity-Related Counseling in Primary Care: 1995–2004. Medical Care. 2007;45(4):322-9.

19. Tsai AG, Wadden TA. Treatment of obesity in primary care practice in the United States: A systematic review. J Gen Intern Med. 2009;24(9):1073-9.

For peer review only - http://bmjopen.bmj.com/site/about/guidelines.xhtml

20. LeBlanc ES, O'Connor E, Whitlock EP, et al. Effectiveness of Primary Care–Relevant Treatments for Obesity in Adults: A Systematic Evidence Review for the U.S. Preventive Services Task Force. Ann Int Med. 2011;155(7):434-47.

21. Jebb SA, Ahern AL, Olson AD, et al. Primary care referral to a commercial provider for weight loss treatment versus standard care: a randomised controlled trial. The Lancet. 2011;378(9801):1485-92.

22. Dixon KJL, Shcherba S, Kipping RR. Weight loss from three commercial providers of NHS primary care slimming on referral in North Somerset: service evaluation. J Public Health. 2012;34(4):555-61.

For peer review only

For peer review only - http://bmjopen.bmj.com/site/about/guidelines.xhtml

Access to weight reduction interventions for overweight and obese patients in UK primary care. Population-based cohort study

Running head: Obesity management in primary care

Article category: Original research

Authors:

Helen P. Booth[1], BSc MSc

A. Toby Prevost[1], BSc MSc PhD

Martin C. Gulliford[1], FFPH FRCP

[1]Department of Primary Care and Public Health Sciences, King's College London, UK

Corresponding author:

Miss Helen P. Booth, Research Associate, Department of Primary Care and Public Health Sciences, King's College London, 7th Floor Capital House, 42 Weston Street, London,

SE1 3QD. Tel: 020 7848 6104. Fax: 020 7848 6620 Email: helen.booth@kcl.ac.uk

References: 21

Tables: 5

Figures: 0

Word count: 3,403

Abstract word count: 271

Supplementary files: 0

Key words: Obesity, Overweight, Primary health care, General practice

For peer review only - http://bmjopen.bmj.com/site/about/guidelines.xhtml

ABSTRACT

Objectives

To investigate access to weight management interventions for overweight and obese patients in primary care

Setting

UK primary care electronic health records

Participants

A cohort of 91,413 overweight and obese patients aged 30 to 100 years was sampled from the Clinical Practice Research Datalink (CPRD). Patients with body mass index (BMI) values ≥25 kg/m^2 recorded between 2005 and 2012 were included. BMI values were categorised using World Health Organization (WHO) criteria.

Interventions

Interventions for body weight management, including advice, referrals and prescription of anti-obesity drugs, were evaluated.

Primary and secondary outcome measures

The rate of body weight management interventions and time to intervention were the main outcomes

Results

Data were analysed for 91,413 patients, mean age 56 years, including 55,094 (60%) overweight and 36,319 (40%) obese, including 4,099 (5%) with morbid obesity. During the study period 90% of overweight patients had no weight management intervention recorded. Intervention was more frequent among obese patients, but 59% of patients with morbid obesity had no intervention recorded. Rates of

For peer review only - http://bmjopen.bmj.com/site/about/guidelines.xhtml

intervention increased with BMI category. In morbid obesity, rates of intervention per 1,000 patient years were: advice, 60.2 (95% CI 51.8 to 70.4); referral, 75.7 (95% CI 69.5 to 82.6); and anti-obesity drugs 89.9 (95% CI 85.0 to 95.2). Weight management interventions were more often accessed by women, older patients, those with co-morbidity and those in deprivation. Follow-up of body weight subsequent to interventions was infrequent.

Conclusions

Limited evidence of weight management interventions in primary care electronic health records may result from poor recording of advice given, but may indicate a lack of patient access to appropriate body weight management interventions in primary care.

ARTICLE SUMMARY

Strengths and limitations of this study

- This study uses primary care electronic health records to investigate the use of weight management interventions in overweight and obese patients
- Lifestyle advice was the most commonly-used intervention in all but morbidly obese patients, where anti-obesity drugs were more frequent
- Patients had to have a BMI value recorded to be included in the study
- Clinicians may be giving advice for weight management but not recording it

For peer review only - http://bmjopen.bmj.com/site/about/guidelines.xhtml

For peer review only

BACKGROUND

Obesity is a leading cause of premature morbidity and mortality worldwide.(1) In the UK, a quarter of adults are obese and up to two thirds are overweight.(2) Primary care represents an important setting in which obese patients may access weight loss interventions. The main strategies for treating obesity are provision of lifestyle advice, referral for weight management, prescription of anti-obesity drugs and, in severe cases, referral for bariatric surgery. Clinical guidelines recommend a stepped approach to weight management depending on the severity of a patient's obesity and whether they have weight-related co-morbidities, with more intensive interventions offered as appropriate. Interventions should be agreed between the clinician and patient, and offered in conjunction with long-term follow-up and continuing care.(3)

Several studies have evaluated the effectiveness of primary care interventions for weight management in obesity, (4-8) but few studies have evaluated how overweight and obese patients are managed in primary care. A survey using data collected in 2000/1, before the UK national guidelines on obesity management were published,(9) found that a fifth of obese patients were offered dietary counselling, less than 5% a referral and 2% anti-obesity medications over an 18 month period. There is no more recent information on the use of interventions for the management of obesity in primary care.

Access to appropriate weight management interventions for overweight and obese patients in primary care is of increasing importance in the context of a national objective to establish a downward trend in obesity among UK adults by 2020.(10)

For peer review only - http://bmjopen.bmj.com/site/about/guidelines.xhtml

For peer review only

This study aimed to evaluate access in terms of recording and utilisation of weight management interventions for overweight and obesity using primary care electronic health records. Interventions were classified as lifestyle advice, referrals for weight management and prescription of anti-obesity drugs.

For peer review only

For peer review only - http://bmjopen.bmj.com/site/about/guidelines.xhtml

METHODS

Data source and cohort definition

A cohort of patients was selected from the Clinical Practice Research Datalink (CPRD), a database of longitudinal patient electronic medical records from UK primary care. CPRD is the largest primary care database in the world, and represents over 5% of the UK population with about 680 practices currently contributing research quality data.(11) The initial cohort was selected as part of a larger project, and comprised a random sample of approximately 300,000 patients who were registered for at least 12 months with a general practice contributing data to CPRD between the 1st January 2005 and 30th April 2012. Equal numbers of men and women were drawn from each year of the study without replacement. Patients were selected who had a body mass index (BMI) record indicating overweight or obesity during the study period. The study was approved by the CPRD Independent Scientific Advisory Committee (ISAC 07_054 and 14_056).

Exposure and outcome definitions

BMI was categorised using the World Health Organization (WHO) categories: overweight (BMI 25-29.9kg/m^2), obese (BMI 30-34.9kg/m^2), severe obesity (BMI 35.0 to 39.9kg/m^2), morbid obesity (BMI≥40kg/m^2). Medical diagnoses of obesity in the medical record were also noted based on the presence of diagnostic codes. Morbidity status was ascertained based on the presence of eleven common conditions associated with obesity, including: type 2 diabetes, coronary heart disease (CHD), stroke, depression, osteoarthritis, back pain, joint problems, cancer, gallbladder disease, asthma and sleep apnoea. Smoking status and socioeconomic

For peer review only - http://bmjopen.bmj.com/site/about/guidelines.xhtml

deprivation were also included as exposures. Socioeconomic deprivation was classified into quintiles using the Index of Multiple Deprivation rank based on patient postcode. Data on deprivation were only available for patients registered at English practices.

Interventions for the management of body weight were identified using medical codes recorded in clinical and referral records, recorded health promotion advice, and prescriptions for anti-obesity drugs. For analysis, weight management interventions were classified into lifestyle advice, referrals for weight management and prescription of anti-obesity drugs. Advice included codes relating to dieting, exercise and weight loss. Relevant referrals included those to community and hospital dieticians, for exercise therapy and for weight management programmes. Prescriptions for three different anti-obesity drugs were included; two of these, sibutramine and rimonabant, have been removed from the UK market because of safety concerns.(12, 13) However, these drugs were in use during the time period investigated and so have been considered in this analysis. The only anti-obesity drug currently licensed in the UK, orlistat, was introduced in 1998 and has been available over the counter as well as by prescription since 2009.(14) Multiple prescriptions of anti-obesity drugs were considered to be a part of the same course of treatment if there was less than 6 months between prescriptions.

Analysis

Person time was analysed following the index date; the first BMI record for overweight or obesity after the 1st January 2005. Patient baseline characteristics were tabulated. The proportion of patients who received weight management

For peer review only - http://bmjopen.bmj.com/site/about/guidelines.xhtml

interventions over the study period was evaluated by BMI category. Time-to-event analysis was used to calculate the rate of intervention utilisation by BMI category and to investigate variables associated with intervention using a multiple-failure multivariable Cox proportional hazards regression model with unordered events.(15) Variation in the use of weight management interventions by GP practice was investigated by calculating the proportion of patients receiving any intervention in the year following the index date. These data were then presented as percentiles of the distribution for all practices. Change in weight from baseline after the implementation of each type of intervention was calculated for up to 5 years of follow-up.

For peer review only

For peer review only - http://bmjopen.bmj.com/site/about/guidelines.xhtml

RESULTS

Of the 300,006 patients in the cohort, 134,697 (45%) had an eligible BMI record. After patients with BMIs lower than 25kg/m^2 were removed, data were analysed for 91,413 patients, with mean age 56 years, including 55,094 (60%) overweight and 36,319 (40%).or obese, including 4,099 (5%) with morbid obesity. Mean age in men and women was 56 years. Patient characteristics on entry to the study are presented in Table 1. At the index date (date of the first relevant BMI record) most patients were overweight (63.9% of men and 56.2% of women); 2.9% of men and 6.3% of women were morbidly obese. A diagnostic code for obesity was recorded for 3.9% of male patients and 6.5% of females. A higher proportion of women were non-smokers, while men were more likely to be former or current smokers.

For peer review only

For peer review only - http://bmjopen.bmj.com/site/about/guidelines.xhtml

Table 1: Characteristics of overweight and obese patients. Figures are frequencies (column percent) unless stated otherwise.

	Men (48,413)	Women (43,000)
**Mean age (SD)**	55.6 (13.9)	56.4 (15.0)
**Body mass index (BMI) category (Kg/m2)**		
Overweight (BMI 25-29.9)	30,950 (63.9)	24,144 (56.2)
Obese (BMI 30-34.9)	12,711 (26.3)	11,364 (26.4)
Severe obesity (BMI 35-39.9)	3,368 (7.0)	4,777 (11.1)
Morbid obesity (BMI ≥40)	1,384 (2.9)	2,715 (6.3)
**Medical code for obesity**	1,876 (3.9)	2,810 (6.5)
**Number of morbidities**		
0	14,810 (30.6)	9,635 (22.4)
1	14,988 (31.0)	11,919 (27.7)
2	10,323 (21.3)	10,237 (23.8)
3 or more	8,292 (17.1)	11,209 (26.1)
**Smoking status**		
Non-smoker	17,415 (36.0)	20,602 (47.9)
Ex-smoker	15,188 (31.4)	9,916 (23.1)
Current smoker	9,359 (19.3)	7,448 (17.3)
Missing smoking status	6,451 (13.3)	5,034 (11.7)
**Index of Multiple Deprivation (IMD) quintile**		
1 – least deprived	11,490 (23.7)	9,229 (21.5)
2	10,850 (22.4)	9,275 (21.6)
3	8,858 (18.3)	7,896 (18.4)
4	7,859 (16.2)	7,413 (17.2)
5 – most deprived	6,310 (13.0)	6,304 (14.7)
Missing IMD	3,046 (6.3)	2,883 (6.7)

For peer review only

For peer review only - http://bmjopen.bmj.com/site/about/guidelines.xhtml

The majority of patients did not receive a weight management intervention during the study period. The proportion of patients by BMI category with each type of intervention recorded on their medical record is given in Table 2. In patients with morbid obesity, 60.0% of men and 58.1% of women had no record of weight management during the 7 years of the study. In patients with non-severe obesity (BMI 30-34.9kg/m^2) the figures were 84.2% and 80.2% respectively. The proportion of patients who received an intervention increased with each additional BMI category. Advice was the most commonly recorded intervention in overweight and obese patients and severely obese men. Drug interventions were the most frequently recorded intervention in morbidly obese patients and severely obese women.

Table 2: Patients who received, or did not receive, a weight management intervention over the study period by gender and BMI category. Figures are frequencies (row percent).

BMI category	Total	Advice	Referral	Drugs	No treatment
		**Men**			
Overweight (BMI 25-29.9)	30,950	1,805 (5.8)	913 (2.9)	86 (0.3)	28,282 (91.4)
Obese (BMI 30-34.9)	12,711	1,129 (8.9)	762 (6.0)	313 (2.5)	10,697 (84.2)
Severe obesity (BMI 35-39.9)	3,368	363 (10.8)	349 (10.4)	333 (9.9)	2,499 (74.2)
Morbid obesity (BMI ≥40)	1,384	168 (12.1)	239 (17.3)	322 (23.3)	831 (60.0)
		**Women**			
Overweight (BMI 25-29.9)	24,144	1,331 (5.5)	762 (3.2)	451 (1.9)	21,794 (90.3)
Obese (BMI 30-34.9)	11,364	925 (8.1)	740 (6.5)	889 (7.8)	9,116 (80.2)
Severe obesity (BMI 35-39.9)	4,777	462 (9.7)	445 (9.3)	671 (14.0)	3,460 (72.4)
Morbid obesity (BMI ≥40)	2,715	284 (10.5)	479 (17.6)	724 (26.7)	1,578 (58.1)

For peer review only - http://bmjopen.bmj.com/site/about/guidelines.xhtml

Rates of intervention are presented in Table 3. Overall, the recorded rates of intervention were highest for advice at 30.3 (95% CI 29.3 to 31.4) per 1,000 person-years. The rate of each intervention type increased in higher BMI categories. The rate of advice was 22.6 (21.6 to 23.8) per 1,000 in overweight patients, and highest at 60.2 (51.8 to 70.4) per 1,000 in morbidly obese patients. In overweight patients, advice was the most commonly used intervention, whereas drug prescription was the most common in morbidly obese patients.

Table 3: Rate of obesity management intervention by body mass index (BMI) category (per 1000 patient years), based on records of advice, referral or obesity drug prescription.

	Advice	Referral	Drugs
**Overall rate**	30.3 (29.3 to 31.4)	20.0 (19.3 to 20.8)	13.9 (13.5 to 14.4)
**Overweight (BMI 25-29.9)**	22.6 (21.6 to 23.8)	11.2 (10.5 to 11.9)	2.9 (2.6 to 3.2)
**Obese (BMI 30-34.9)**	36.4 (34.4 to 38.6)	23.7 (22.3 to 25.2)	15.7 (14.7 to 16.7)
**Severe obesity (BMI 35-39.9)**	47.2 (42.7 to 52.3)	38.4 (35.4 to 41.7)	41.5 (39.0 to 44.1)
**Morbid obesity (BMI ≥40)**	60.2 (51.8 to 70.4)	75.7 (69.5 to 82.6)	89.9 (85.0 to 95.2)

For peer review only - http://bmjopen.bmj.com/site/about/guidelines.xhtml

The multivariable analysis identified BMI category as the strongest predictor of weight-loss intervention, with a hazard ratio (HR) of 1.68 (95% CI 1.60 to 1.76) for obesity and 3.67 (95% CI 3.45 to 3.92) for morbid obesity (Table 4). Increasing age, type 2 diabetes and depression were also strong predictors of patients receiving a weight loss intervention. Female gender, being a former smoker and socioeconomic deprivation were associated with treatment for overweight and obesity.

Table 4: Cox proportional hazards model investigating time to multiple weight management interventions after a record of overweight or obesity.

	Patients receiving weight management intervention (n)	Total patients (N)	Hazard ratio	95% confidence interval	P value
**Age (decades)**	-	-	1.42	1.27 to 1.58	<0.001
**Age squared**	-	-	0.97	0.96 to 0.98	<0.001
**Gender**					
Male	6,104	48,413	1.00	-	-
Female	7,054	43,000	1.14	1.10 to 1.19	<0.001
**Body mass index (BMI) category***					
Overweight (BMI 25-29.9)	5,019	50,075	1.00	-	-
Obese (BMI 30-34.9)	4,263	19,812	1.68	1.60 to 1.76	<0.001
Severe obesity (BMI 35-39.9)	2,186	5,959	2.36	2.23 to 2.50	<0.001
Morbid obesity (BMI ≥40)	1,690	2,409	3.67	3.45 to 3.91	<0.001
**Smoking status**					
Non-smoker	5,441	32,576	1.00	-	-
Former smoker	3,962	24,142	1.11	1.06 to 1.16	<0.001
Current smoker	2,530	14,277	0.99	0.94 to 1.05	0.823
Missing smoking status	1,225	10,260	0.82	0.77 to 0.88	<0.001
**Index of Multiple Deprivation quintile**					
1 - least deprived	2,564	18,155	1.00	-	-
2	2,490	17,635	0.94	0.88 to 1.00	0.054
3	2,511	14,243	1.20	1.12 to 1.29	<0.001
4	2,413	12,859	1.13	1.06 to 1.21	<0.001
5 – most deprived	2,277	10,337	1.24	1.15 to 1.32	<0.001
Missing IMD	903	5,026	1.04	0.95 to 1.13	0.395
**Coronary heart disease (CHD)**	1,993	9,669	1.24	1.16 to 1.31	<0.001
**Stroke**	535	2,603	1.09	0.98 to 1.21	0.116
**Type 2 diabetes**	4,401	12,884	1.83	1.75 to 1.92	<0.001
**Depression**	6,385	31,573	1.33	1.28 to 1.39	<0.001

* BMI group at baseline. Patients could change BMI category in the analysis so intervention may have been delivered when patients had changed BMI category

For peer review only - http://bmjopen.bmj.com/site/about/guidelines.xhtml

There was substantial variation between practices in the recording of obesity management interventions (see Table 5). The median proportion of obese and overweight patients receiving a weight management intervention during the study was 12% (Interquartile range (IQR) 7 to 19). A maximum of 91% overweight or obese patients in a practice had an intervention recorded. Follow-up measurements of body weight after intervention were most frequent in patients who had a referral, with 34.1% of patients having a weight measurement in the first year. In contrast, 20.7% of patients had a follow-up weight measurement in the first year after advice and 24.3% after a drug prescription. No trend in weight change was observed in patients up to five years after any of the three intervention types investigated.

Table 5: Use of weight management interventions in general practices. Figures are percentiles among 491 GP practices representing the proportion of patients in the practice receiving interventions.

	Minimum	10th percentile	25th percentile	Median	75th percentile	90th percentile	Maximum
**Patients receiving any intervention (%)**	0	4	7	12	19	28	91
**Patients receiving advice (%)**	0	0	0	3	9	18	91
**Patients receiving a referral (%)**	0	0	1	3	7	13	50
**Patients receiving anti-obesity drugs (%)**	0	0	2	4	6	9	33

NB: different practices may occupy centiles for different measures

For peer review only - http://bmjopen.bmj.com/site/about/guidelines.xhtml

DISCUSSION

Summary

Analysis of primary care electronic health records reveals that the use of weight management interventions in primary care for the treatment of overweight and obesity were infrequent between 2005 and 2012. The likelihood of intervention was strongly associated with BMI category. However, 60% of men and 58% of women with morbid obesity did not have any record of receiving weight management in primary care, with higher proportions noted in lower BMI categories. Variation in obesity management between general practices was evident, with many practices not recording any intervention. These results might be a consequence of poor documentation of advice given, but might also indicate a lack of patient access to appropriate body weight management interventions in primary care due to a lack of clinician awareness or confidence in treating obesity.

There was some evidence body weight management was tailored to obesity category with more frequent utilisation of anti-obesity drugs in patients who were in higher obesity categories and advice used more commonly in overweight patients. BMI category was the strongest predictor of a patient receiving weight management interventions, with rates over 3-times higher in morbid obesity than in overweight. Female gender, increasing age, socioeconomic deprivation and co-morbidities were also associated with greater use of weight management interventions.

Follow-up values for body weight after a recorded weight management intervention were limited. Monitoring of body weight in primary care is generally opportunistic and depends on patients attending the practice and having a weight measurement recorded. However,

For peer review only - http://bmjopen.bmj.com/site/about/guidelines.xhtml

the relatively high levels of co-morbidity in patients in this cohort, including those that require long-term management such as type 2 diabetes, suggest that consultations are likely to be regular. While follow-up weight measurements did not show any change in weight after intervention; these results are very vulnerable to information bias.

Comparison with the literature

One other UK-based study investigated using of primary care interventions for the treatment of obesity.(9) The Counterweight report identified that 20% of patients received advice, 4% referrals and 2% anti-obesity drugs based on a review of 100 obese patients medical records over an 18-month period in 2000-01. We identified a smaller proportion of patients receiving advice and a higher proportion having a referral or drug prescription over a longer time period. It was not clear how obese participants were selected in the Counterweight study. Other differences between the present study and the Counterweight paper include a larger sample size and inclusion of overweight patients. However, the results suggest that prescribing of anti-obesity drugs has increased in the last 15 years. Increased use of anti-obesity drugs between 1998, when they were first introduced in the UK, and 2005 has been reported elsewhere.(16)

A decline in lifestyle advice and counselling for weight loss given to obese patients over the last 10 years has been also been reported in studies from the US. Reasons behind this reduction, despite increasing obesity levels, include poor recording of advice, lack of time in consultations, pessimism regarding potential success of weight loss attempts and increased use of medications to treat obesity-related risk factors and disease (17, 18) and, perhaps, normalisation of excessive body weight. Although the evidence from the current study is not sufficient to conclude that a reduction in advice for weight management has

For peer review only - http://bmjopen.bmj.com/site/about/guidelines.xhtml

occurred, some of the explanations attributed to lowered rates in the US are likely to be applicable in the UK.

Strengths and limitations

This study design had the advantage of a large population-based sample taken from different regions of the UK. However, it is likely that not all weight management interventions, particularly lifestyle advice, were captured in the electronic health record. Brief advice may be given to patients but not recorded by clinicians, which could have led to an underestimation of intervention rates. This is less likely to be an issue with referrals and drug prescribing. Furthermore, the patients included in this sample were selected on the basis of having a BMI record indicating that they were overweight or obese. This may have introduced a selection bias as these patients have been identified as having a weight problem by a clinician. Patients who are obese but do not have a record of weight status in their medical record may or may not be receiving weight management interventions differently from those who have been diagnosed.

Implications for practice and future research

The results of this study suggest that primary-care interventions given to patients with the aim of reducing weight are under-utilised, and that follow-up to determine their success is poor. It is possible that rates have been under-estimated through a lack of formal recording in medical records. However, the growing burden of obesity on primary healthcare services and lack of long-term follow-up on the effectiveness of these treatments supports the use of structured recording of interventions for weight management and subsequent follow-up. This is particularly true given the heterogeneity of results from weight loss studies included in reviews of the effectiveness of primary-care interventions for obesity

For peer review only - http://bmjopen.bmj.com/site/about/guidelines.xhtml

and the need for further evidence specific to patient sub-groups e.g. those with co-morbidities (8, 19, 20). Primary care referrals to commercial weight loss programmes have been found to be effective in trials.(21-22) Although this type of referral wasn't included in the present study, an analysis using primary care data could be valuable. Data in CPRD are not specific enough to permit this at present. Consistency of public health messages on the health risks associated with obesity should be promoted in primary care where clinicians have the opportunity to reach a large number of patients and utilise preventive as well as reactive treatment strategies.

Study funding: This study was supported by the UK National Prevention Research Initiative whose funding partners include the Alzheimer's Research Trust; Alzheimer's Society; Biotechnology and Biological Sciences Research Council; British Heart Foundation; Cancer Research UK; Chief Scientist Office, Scottish Government Health Directorate; Department of Health; Diabetes UK; Economic and Social Research Council; Engineering and Physical Sciences Research Council; Health & Social Care Research & Development Office for Northern Ireland; Medical Research Council; The Stroke Association; Welsh Assembly Government; and World Cancer Research Fund.The views expressed are those of the authors alone.

Competing interests: None declared

Contributions: HB & MG designed the study. TP & MG advised on the conduct of the data analysis. HB conducted the analysis and drafted the paper. MG & TP contributed to interpretation of the results. All authors commented and approved the manuscript. HB is guarantor.

For peer review only - http://bmjopen.bmj.com/site/about/guidelines.xhtml

REFERENCES

1. Lopez AD, Mathers CD, Ezzati M, editors. Global burden of disease and risk factors. Washington: The World Bank & Oxford University Press; 2006.

2. Moody A. Chapter 10: Adult anthropometric measures, overweight and obesity. London, UK: Health and Social Care Information Centre, 2013.

3. National Institute for Health and Care Excellence. Obesity: Identification, assessment and management of overweight and obesity in children, young people and adults (draft for consultation). London, UK: National Institute for Health and Care Excellence, 2014 Contract No.: CG43.

4. Ara R, Blake L, Gray L, et al. What is the clinical effectiveness and cost-effectiveness of using drugs in treating obese patients in primary care? A systematic review. Health Technology Assessment. 2012;16(5).

5. Avenell A, Broom J, Brown TJ, et al. Systematic review of the long-term effects and economic consequences of treatments for obesity and implications for health improvement. Health Technology Assessment. 2004;8(21).

6. Picot J, Jones J, Colquitt J, et al. Weight Loss Surgery for Mild to Moderate Obesity: A Systematic Review and Economic Evaluation. Obesity Surgery. 2012;22(9):1496-506.

7. Picot J, Jones J, Colquitt JL, et al. The clinical effectiveness and cost-effectiveness of bariatric (weight loss) surgery for obesity: a systematic review and economic evaluation. Health Technology Assessment. 2009;13(41):215-357.

8. Booth HP, Prevost AT, Wright AJ, et al. (in press) Effectiveness of behavioral weight loss interventions delivered in a primary care setting: A systematic review and meta-analysis. Family Practice. 2014.

9. Counterweight Project Team. Current approaches to obesity management in UK primary care: the Counterweight programme. J Hum Nutr Diet. 2004;17:7.

For peer review only - http://bmjopen.bmj.com/site/about/guidelines.xhtml

10. Department of Health. Healthy Lives, Healthy People: a call to action on obesity. London: HM Government, 2011 Contract No.: 16166.

11. Williams T, van Staa T, Puri S, et al. Recent advances in the utility and use of the General Practice Research Database as an example of a UK Primary Care Data resource. Therapeutic Advances in Drug Safety. 2012;3(2):89-99.

12. Medicines and Healthcare Products Regulatory Agency. Suspension of sibutramine (Reductil) 2010 [19/03/2014]. Available from: http://www.mhra.gov.uk/NewsCentre/CON068470.

13. Medicines and Healthcare Products Regulatory Agency. Rimonabant: European suspension of marketing authorisation 2008 [cited 26/08/2014]. Available from: http://www.mhra.gov.uk/Safetyinformation/DrugSafetyUpdate/CON087927.

14. Kang JG, Park C-Y. Anti-Obesity Drugs: A Review about Their Effects and Safety. Diabetes Metab J. 2012;36(1):13–25.

15. Cleves M, StataCorp. Analysis of multiple failure-time data with Stata 2009 [cited 10/09/2014]. Available from: http://www.stata.com/support/faqs/statistics/multiple-failure-time-data/.

16. Srishanmuganathan J, Patel H, Car J, Majeed A. National trends in the use and costs of anti-obesity medications in England 1998–2005. J Public Health. 2007;29(2):199-202.

17. Yates EA, Macpherson AK, Kuk JL. Secular Trends in the Diagnosis and Treatment of Obesity Among US Adults in the Primary Care Setting. Obesity. 2012;20(9):1909-14.

18. McAlpine DDW, A R. Trends in Obesity-Related Counseling in Primary Care: 1995–2004. Medical Care. 2007;45(4):322-9.

19. Tsai AG, Wadden TA. Treatment of obesity in primary care practice in the United States: A systematic review. J Gen Intern Med. 2009;24(9):1073-9.

For peer review only - http://bmjopen.bmj.com/site/about/guidelines.xhtml

20. LeBlanc ES, O'Connor E, Whitlock EP, et al. Effectiveness of Primary Care–Relevant Treatments for Obesity in Adults: A Systematic Evidence Review for the U.S. Preventive Services Task Force. Ann Int Med. 2011;155(7):434-47.

21. Jebb SA, Ahern AL, Olson AD, et al. Primary care referral to a commercial provider for weight loss treatment versus standard care: a randomised controlled trial. The Lancet. 2011;378(9801):1485-92.

22. Dixon KJL, Shcherba S, Kipping RR. Weight loss from three commercial providers of NHS primary care slimming on referral in North Somerset: service evaluation. J Public Health. 2012;34(4):555-61.

For peer review only

For peer review only - http://bmjopen.bmj.com/site/about/guidelines.xhtml

STROBE Statement—checklist of items that should be included in reports of observational studies

	Item No	Recommendation
**Title and abstract**	1	(*a*) Indicate the study's design with a commonly used term in the title or the abstract [p1]
		(*b*) Provide in the abstract an informative and balanced summary of what was done and what was found [p2]
**Introduction**		
Background/rationale	2	Explain the scientific background and rationale for the investigation being reported [p4, 2nd para]
Objectives	3	State specific objectives, including any prespecified hypotheses [abstract, p2 and p5]
**Methods**		
Study design	4	Present key elements of study design early in the paper [p6]
Setting	5	Describe the setting, locations, and relevant dates, including periods of recruitment, exposure, follow-up, and data collection [p6]
Participants	6	(*a*) *Cohort study*—Give the eligibility criteria, and the sources and methods of selection of participants. Describe methods of follow-up [p6]<br>*Case-control study*—Give the eligibility criteria, and the sources and methods of case ascertainment and control selection. Give the rationale for the choice of cases and controls<br>*Cross-sectional study*—Give the eligibility criteria, and the sources and methods of selection of participants
		(*b*) *Cohort study*—For matched studies, give matching criteria and number of exposed and unexposed<br>*Case-control study*—For matched studies, give matching criteria and the number of controls per case
Variables	7	Clearly define all outcomes, exposures, predictors, potential confounders, and effect modifiers. Give diagnostic criteria, if applicable [p6 – exposure and outcome definitions]
Data sources/ measurement	8*	For each variable of interest, give sources of data and details of methods of assessment (measurement). Describe comparability of assessment methods if there is more than one group [p6 –7]
Bias	9	Describe any efforts to address potential sources of bias
Study size	10	Explain how the study size was arrived at
Quantitative variables	11	Explain how quantitative variables were handled in the analyses. If applicable, describe which groupings were chosen and why [p6 – exposure and outcome definitions]
Statistical methods	12	(*a*) Describe all statistical methods, including those used to control for confounding [p7-8]
		(*b*) Describe any methods used to examine subgroups and interactions
		(*c*) Explain how missing data were addressed
		(*d*) *Cohort study*—If applicable, explain how loss to follow-up was addressed<br>*Case-control study*—If applicable, explain how matching of cases and controls was addressed<br>*Cross-sectional study*—If applicable, describe analytical methods taking account of

For peer review only - http://bmjopen.bmj.com/site/about/guidelines.xhtml

For peer review only

	sampling strategy
	(*e*) Describe any sensitivity analyses

Continued on next page

For peer review only

**Results**		
Participants	13*	(a) Report numbers of individuals at each stage of study—eg numbers potentially eligible, examined for eligibility, confirmed eligible, included in the study, completing follow-up, and analysed
		(b) Give reasons for non-participation at each stage
		(c) Consider use of a flow diagram
Descriptive data	14*	(a) Give characteristics of study participants (eg demographic, clinical, social) and information on exposures and potential confounders [p9 & Table 1]
		(b) Indicate number of participants with missing data for each variable of interest [Table 1]
		(c) *Cohort study*—Summarise follow-up time (eg, average and total amount)
Outcome data	15*	*Cohort study*—Report numbers of outcome events or summary measures over time [p11-12]
		Case-control study—Report numbers in each exposure category, or summary measures of exposure
		Cross-sectional study—Report numbers of outcome events or summary measures
Main results	16	(*a*) Give unadjusted estimates and, if applicable, confounder-adjusted estimates and their precision (eg, 95% confidence interval). Make clear which confounders were adjusted for and why they were included [throughout results]
		(*b*) Report category boundaries when continuous variables were categorized [p6 – BMI]
		(*c*) If relevant, consider translating estimates of relative risk into absolute risk for a meaningful time period
Other analyses	17	Report other analyses done—eg analyses of subgroups and interactions, and sensitivity analyses
**Discussion**		
Key results	18	Summarise key results with reference to study objectives [p15]
Limitations	19	Discuss limitations of the study, taking into account sources of potential bias or imprecision. Discuss both direction and magnitude of any potential bias [p17]
Interpretation	20	Give a cautious overall interpretation of results considering objectives, limitations, multiplicity of analyses, results from similar studies, and other relevant evidence [p17]
Generalisability	21	Discuss the generalisability (external validity) of the study results [p17]
**Other information**		
Funding	22	Give the source of funding and the role of the funders for the present study and, if applicable, for the original study on which the present article is based [p18]

*Give information separately for cases and controls in case-control studies and, if applicable, for exposed and unexposed groups in cohort and cross-sectional studies.

Note: An Explanation and Elaboration article discusses each checklist item and gives methodological background and published examples of transparent reporting. The STROBE checklist is best used in conjunction with this article (freely available on the Web sites of PLoS Medicine at http://www.plosmedicine.org/, Annals of Internal Medicine at http://www.annals.org/, and Epidemiology at http://www.epidem.com/). Information on the STROBE Initiative is available at www.strobe-statement.org.

For peer review only

BMJ Open

Access to weight reduction interventions for overweight and obese patients in UK primary care. Population-based cohort study

Journal:	*BMJ Open*
Manuscript ID:	bmjopen-2014-006642.R2
Article Type:	Research
Date Submitted by the Author:	14-Nov-2014
Complete List of Authors:	Booth, Helen; King's College London, Primary Care and Public Health Sciences<br>Prevost, A.; King's College London, Primary Care and Public Health Sciences<br>Gulliford, Martin; King's College London, Primary Care and Public Health Sciences
**Primary Subject Heading**:	General practice / Family practice
Secondary Subject Heading:	Public health
Keywords:	PRIMARY CARE, PUBLIC HEALTH, EPIDEMIOLOGY

SCHOLARONE™
Manuscripts

For peer review only

Access to weight reduction interventions for overweight and obese patients in UK primary care. Population-based cohort study

Running head: Obesity management in primary care

Article category: Original research

Authors:
Helen P. Booth[1], BSc MSc
A. Toby Prevost[1], BSc MSc PhD
Martin C. Gulliford[1], FFPH FRCP
[1]Department of Primary Care and Public Health Sciences, King's College London, UK

Corresponding author:
Miss Helen P. Booth, Research Associate, Department of Primary Care and Public Health Sciences, King's College London, 7th Floor Capital House, 42 Weston Street, London,
SE1 3QD. Tel: 020 7848 6104. Fax: 020 7848 6620 Email: helen.booth@kcl.ac.uk

References: 21
Tables: 5
Figures: 0
Word count: 3,403
Abstract word count: 271
Supplementary files: 0
Key words: Obesity, Overweight, Primary health care, General practice

For peer review only

For peer review only - http://bmjopen.bmj.com/site/about/guidelines.xhtml

ABSTRACT

Objectives

To investigate access to weight management interventions for overweight and obese patients in primary care

Setting

UK primary care electronic health records

Participants

A cohort of 91,413 overweight and obese patients aged 30 to 100 years was sampled from the Clinical Practice Research Datalink (CPRD). Patients with body mass index (BMI) values ≥25 kg/m^2 recorded between 2005 and 2012 were included. BMI values were categorised using World Health Organization (WHO) criteria.

Interventions

Interventions for body weight management, including advice, referrals and prescription of anti-obesity drugs, were evaluated.

Primary and secondary outcome measures

The rate of body weight management interventions and time to intervention were the main outcomes

Results

Data were analysed for 91,413 patients, mean age 56 years, including 55,094 (60%) overweight and 36,319 (40%) obese, including 4,099 (5%) with morbid obesity. During the study period 90% of overweight patients had no weight management intervention recorded. Intervention was more frequent among obese patients, but 59% of patients with morbid obesity had no intervention recorded. Rates of

For peer review only - http://bmjopen.bmj.com/site/about/guidelines.xhtml

intervention increased with BMI category. In morbid obesity, rates of intervention per 1,000 patient years were: advice, 60.2 (95% CI 51.8 to 70.4); referral, 75.7 (95% CI 69.5 to 82.6); and anti-obesity drugs 89.9 (95% CI 85.0 to 95.2). Weight management interventions were more often accessed by women, older patients, those with co-morbidity and those in deprivation. Follow-up of body weight subsequent to interventions was infrequent.

Conclusions

Limited evidence of weight management interventions in primary care electronic health records may result from poor recording of advice given, but may indicate a lack of patient access to appropriate body weight management interventions in primary care.

ARTICLE SUMMARY

Strengths and limitations of this study

- This study uses primary care electronic health records to investigate the use of weight management interventions in overweight and obese patients
- Lifestyle advice was the most commonly-used intervention in all but morbidly obese patients, where anti-obesity drugs were more frequent
- Patients had to have a BMI value recorded to be included in the study
- Clinicians may be giving advice for weight management but not recording it

For peer review only

For peer review only - http://bmjopen.bmj.com/site/about/guidelines.xhtml

BACKGROUND

Obesity is a leading cause of premature morbidity and mortality worldwide.(1) In the UK, a quarter of adults are obese and up to two thirds are overweight.(2) Primary care represents an important setting in which obese patients may access weight loss interventions. The main strategies for treating obesity are provision of lifestyle advice, referral for weight management, prescription of anti-obesity drugs and, in severe cases, referral for bariatric surgery. Clinical guidelines recommend a stepped approach to weight management depending on the severity of a patient's obesity and whether they have weight-related co-morbidities, with more intensive interventions offered as appropriate. Interventions should be agreed between the clinician and patient, and offered in conjunction with long-term follow-up and continuing care.(3)

Several studies have evaluated the effectiveness of primary care interventions for weight management in obesity, (4-8) but few studies have evaluated how overweight and obese patients are managed in primary care. A survey using data collected in 2000/1, before the UK national guidelines on obesity management were published,(9) found that a fifth of obese patients were offered dietary counselling, less than 5% a referral and 2% anti-obesity medications over an 18 month period. There is no more recent information on the use of interventions for the management of obesity in primary care.

Access to appropriate weight management interventions for overweight and obese patients in primary care is of increasing importance in the context of a national objective to establish a downward trend in obesity among UK adults by 2020.(10)

For peer review only - http://bmjopen.bmj.com/site/about/guidelines.xhtml

For peer review only

This study aimed to evaluate access in terms of recording and utilisation of weight management interventions for overweight and obesity using primary care electronic health records. Interventions were classified as lifestyle advice, referrals for weight management and prescription of anti-obesity drugs.

For peer review only

For peer review only - http://bmjopen.bmj.com/site/about/guidelines.xhtml

METHODS

Data source and cohort definition

A cohort of patients was selected from the Clinical Practice Research Datalink (CPRD), a database of longitudinal patient electronic medical records from UK primary care. CPRD is the largest primary care database in the world, and represents over 5% of the UK population with about 680 practices currently contributing research quality data.(11) The initial cohort was selected as part of a larger project, and comprised a random sample of approximately 300,000 patients who were registered for at least 12 months with a general practice contributing data to CPRD between the 1st January 2005 and 30th April 2012. Equal numbers of men and women were drawn from each year of the study without replacement. Patients were selected who had a body mass index (BMI) record indicating overweight or obesity during the study period. The study was approved by the CPRD Independent Scientific Advisory Committee (ISAC 07_054 and 14_056).

Exposure and outcome definitions

BMI was categorised using the World Health Organization (WHO) categories: overweight (BMI 25-29.9kg/m^2), obese (BMI 30-34.9kg/m^2), severe obesity (BMI 35.0 to 39.9kg/m^2), morbid obesity (BMI≥40kg/m^2). Medical diagnoses of obesity in the medical record were also noted based on the presence of diagnostic codes. Morbidity status was ascertained based on the presence of eleven common conditions associated with obesity, including: type 2 diabetes, coronary heart disease (CHD), stroke, depression, osteoarthritis, back pain, joint problems, cancer, gallbladder disease, asthma and sleep apnoea. Smoking status and socioeconomic

For peer review only - http://bmjopen.bmj.com/site/about/guidelines.xhtml

deprivation were also included as exposures. Socioeconomic deprivation was classified into quintiles using the Index of Multiple Deprivation rank based on patient postcode. Data on deprivation were only available for patients registered at English practices.

Interventions for the management of body weight were identified using medical codes recorded in clinical and referral records, recorded health promotion advice, and prescriptions for anti-obesity drugs. For analysis, weight management interventions were classified into lifestyle advice, referrals for weight management and prescription of anti-obesity drugs. Advice included codes relating to dieting, exercise and weight loss. Relevant referrals included those to community and hospital dieticians, for exercise therapy and for weight management programmes. Prescriptions for three different anti-obesity drugs were included; two of these, sibutramine and rimonabant, have been removed from the UK market because of safety concerns.(12, 13) However, these drugs were in use during the time period investigated and so have been considered in this analysis. The only anti-obesity drug currently licensed in the UK, orlistat, was introduced in 1998 and has been available over the counter as well as by prescription since 2009.(14) Multiple prescriptions of anti-obesity drugs were considered to be a part of the same course of treatment if there was less than 6 months between prescriptions.

Analysis

Person time was analysed following the index date; the first BMI record for overweight or obesity after the 1st January 2005. Patient baseline characteristics were tabulated. The proportion of patients who received weight management

For peer review only - http://bmjopen.bmj.com/site/about/guidelines.xhtml

interventions over the study period was evaluated by BMI category. Time-to-event analysis was used to calculate the rate of intervention utilisation by BMI category and to investigate variables associated with intervention using a multiple-failure multivariable Cox proportional hazards regression model with unordered events.(15) Variation in the use of weight management interventions by GP practice was investigated by calculating the proportion of patients receiving any intervention in the year following the index date. These data were then presented as percentiles of the distribution for all practices. Change in weight from baseline after the implementation of each type of intervention was calculated for up to 5 years of follow-up.

For peer review only

For peer review only - http://bmjopen.bmj.com/site/about/guidelines.xhtml

RESULTS

Of the 300,006 patients in the cohort, 134,697 (45%) had an eligible BMI record. After patients with BMIs lower than 25kg/m^2 were removed, data were analysed for 91,413 patients, with mean age 56 years, including 55,094 (60%) overweight and 36,319 (40%).or obese, including 4,099 (5%) with morbid obesity. Mean age in men and women was 56 years. Patient characteristics on entry to the study are presented in Table 1. At the index date (date of the first relevant BMI record) most patients were overweight (63.9% of men and 56.2% of women); 2.9% of men and 6.3% of women were morbidly obese. A diagnostic code for obesity was recorded for 3.9% of male patients and 6.5% of females. A higher proportion of women were non-smokers, while men were more likely to be former or current smokers.

For peer review only

For peer review only - http://bmjopen.bmj.com/site/about/guidelines.xhtml

Table 1: Characteristics of overweight and obese patients. Figures are frequencies (column percent) unless stated otherwise.

	Men (48,413)	Women (43,000)
**Mean age (SD)**	55.6 (13.9)	56.4 (15.0)
**Body mass index (BMI) category (Kg/m2)**		
Overweight (BMI 25-29.9)	30,950 (63.9)	24,144 (56.2)
Obese (BMI 30-34.9)	12,711 (26.3)	11,364 (26.4)
Severe obesity (BMI 35-39.9)	3,368 (7.0)	4,777 (11.1)
Morbid obesity (BMI ≥40)	1,384 (2.9)	2,715 (6.3)
**Medical code for obesity**	1,876 (3.9)	2,810 (6.5)
**Number of morbidities**		
0	14,810 (30.6)	9,635 (22.4)
1	14,988 (31.0)	11,919 (27.7)
2	10,323 (21.3)	10,237 (23.8)
3 or more	8,292 (17.1)	11,209 (26.1)
**Smoking status**		
Non-smoker	17,415 (36.0)	20,602 (47.9)
Ex-smoker	15,188 (31.4)	9,916 (23.1)
Current smoker	9,359 (19.3)	7,448 (17.3)
Missing smoking status	6,451 (13.3)	5,034 (11.7)
**Index of Multiple Deprivation (IMD) quintile**		
1 – least deprived	11,490 (23.7)	9,229 (21.5)
2	10,850 (22.4)	9,275 (21.6)
3	8,858 (18.3)	7,896 (18.4)
4	7,859 (16.2)	7,413 (17.2)
5 – most deprived	6,310 (13.0)	6,304 (14.7)
Missing IMD	3,046 (6.3)	2,883 (6.7)

For peer review only

For peer review only - http://bmjopen.bmj.com/site/about/guidelines.xhtml

The majority of patients did not receive a weight management intervention during the study period. The proportion of patients by BMI category with each type of intervention recorded on their medical record is given in Table 2. In patients with morbid obesity, 60.0% of men and 58.1% of women had no record of weight management during the 7 years of the study. In patients with non-severe obesity (BMI 30-34.9kg/m^2) the figures were 84.2% and 80.2% respectively. The proportion of patients who received an intervention increased with each additional BMI category. Advice was the most commonly recorded intervention in overweight and obese patients and severely obese men. Drug interventions were the most frequently recorded intervention in morbidly obese patients and severely obese women.

Table 2: Patients who received, or did not receive, a weight management intervention over the study period by gender and BMI category. Figures are frequencies (row percent).

BMI category	Total	Advice	Referral	Drugs	No treatment
		**Men**			
Overweight (BMI 25-29.9)	30,950	1,805 (5.8)	913 (2.9)	86 (0.3)	28,282 (91.4)
Obese (BMI 30-34.9)	12,711	1,129 (8.9)	762 (6.0)	313 (2.5)	10,697 (84.2)
Severe obesity (BMI 35-39.9)	3,368	363 (10.8)	349 (10.4)	333 (9.9)	2,499 (74.2)
Morbid obesity (BMI ≥40)	1,384	168 (12.1)	239 (17.3)	322 (23.3)	831 (60.0)
		**Women**			
Overweight (BMI 25-29.9)	24,144	1,331 (5.5)	762 (3.2)	451 (1.9)	21,794 (90.3)
Obese (BMI 30-34.9)	11,364	925 (8.1)	740 (6.5)	889 (7.8)	9,116 (80.2)
Severe obesity (BMI 35-39.9)	4,777	462 (9.7)	445 (9.3)	671 (14.0)	3,460 (72.4)
Morbid obesity (BMI ≥40)	2,715	284 (10.5)	479 (17.6)	724 (26.7)	1,578 (58.1)

For peer review only - http://bmjopen.bmj.com/site/about/guidelines.xhtml

Rates of intervention are presented in Table 3. Overall, the recorded rates of intervention were highest for advice at 30.3 (95% CI 29.3 to 31.4) per 1,000 person-years. The rate of each intervention type increased in higher BMI categories. The rate of advice was 22.6 (21.6 to 23.8) per 1,000 in overweight patients, and highest at 60.2 (51.8 to 70.4) per 1,000 in morbidly obese patients. In overweight patients, advice was the most commonly used intervention, whereas drug prescription was the most common in morbidly obese patients.

Table 3: Rate of obesity management intervention by body mass index (BMI) category (per 1000 patient years), based on records of advice, referral or obesity drug prescription.

	Advice	Referral	Drugs
**Overall rate**	30.3 (29.3 to 31.4)	20.0 (19.3 to 20.8)	13.9 (13.5 to 14.4)
**Overweight (BMI 25-29.9)**	22.6 (21.6 to 23.8)	11.2 (10.5 to 11.9)	2.9 (2.6 to 3.2)
**Obese (BMI 30-34.9)**	36.4 (34.4 to 38.6)	23.7 (22.3 to 25.2)	15.7 (14.7 to 16.7)
**Severe obesity (BMI 35-39.9)**	47.2 (42.7 to 52.3)	38.4 (35.4 to 41.7)	41.5 (39.0 to 44.1)
**Morbid obesity (BMI ≥40)**	60.2 (51.8 to 70.4)	75.7 (69.5 to 82.6)	89.9 (85.0 to 95.2)

For peer review only - http://bmjopen.bmj.com/site/about/guidelines.xhtml

The multivariable analysis identified BMI category as the strongest predictor of weight-loss intervention, with a hazard ratio (HR) of 1.68 (95% CI 1.60 to 1.76) for obesity and 3.67 (95% CI 3.45 to 3.92) for morbid obesity (Table 4). Increasing age, type 2 diabetes and depression tended to be associated with receiving a weight loss intervention. Female gender, being a former smoker and socioeconomic deprivation were associated with treatment for overweight and obesity.

Table 4: Cox proportional hazards model investigating time to multiple weight management interventions after a record of overweight or obesity.

	Patients receiving weight management intervention (n)	Total patients (N)	Hazard ratio	95% confidence interval	P value
**Age (decades)**	-	-	1.42	1.27 to 1.58	<0.001
**Age squared**	-	-	0.97	0.96 to 0.98	<0.001
**Gender**					
Male	6,104	48,413	1.00	-	-
Female	7,054	43,000	1.14	1.10 to 1.19	<0.001
**Body mass index (BMI) category***					
Overweight (BMI 25-29.9)	5,019	50,075	1.00	-	-
Obese (BMI 30-34.9)	4,263	19,812	1.68	1.60 to 1.76	<0.001
Severe obesity (BMI 35-39.9)	2,186	5,959	2.36	2.23 to 2.50	<0.001
Morbid obesity (BMI ≥40)	1,690	2,409	3.67	3.45 to 3.91	<0.001
**Smoking status**					
Non-smoker	5,441	32,576	1.00	-	-
Former smoker	3,962	24,142	1.11	1.06 to 1.16	<0.001
Current smoker	2,530	14,277	0.99	0.94 to 1.05	0.823
Missing smoking status	1,225	10,260	0.82	0.77 to 0.88	<0.001
**Index of Multiple Deprivation quintile**					
1 - least deprived	2,564	18,155	1.00	-	-
2	2,490	17,635	0.94	0.88 to 1.00	0.054
3	2,511	14,243	1.20	1.12 to 1.29	<0.001
4	2,413	12,859	1.13	1.06 to 1.21	<0.001
5 – most deprived	2,277	10,337	1.24	1.15 to 1.32	<0.001
Missing IMD	903	5,026	1.04	0.95 to 1.13	0.395
**Coronary heart disease (CHD)**	1,993	9,669	1.24	1.16 to 1.31	<0.001
**Stroke**	535	2,603	1.09	0.98 to 1.21	0.116
**Type 2 diabetes**	4,401	12,884	1.83	1.75 to 1.92	<0.001
**Depression**	6,385	31,573	1.33	1.28 to 1.39	<0.001

* BMI group at baseline. Patients could change BMI category in the analysis so intervention may have been delivered when patients had changed BMI category

For peer review only - http://bmjopen.bmj.com/site/about/guidelines.xhtml

For peer review only

There was substantial variation between practices in the recording of obesity management interventions (see Table 5). The median proportion of obese and overweight patients receiving a weight management intervention during the study was 12% (Interquartile range (IQR) 7 to 19). A maximum of 91% overweight or obese patients in a practice had an intervention recorded. Follow-up measurements of body weight after intervention were most frequent in patients who had a referral, with 34.1% of patients having a weight measurement in the first year. In contrast, 20.7% of patients had a follow-up weight measurement in the first year after advice and 24.3% after a drug prescription. No trend in weight change was observed in patients up to five years after any of the three intervention types investigated.

Table 5: Use of weight management interventions in general practices. Figures are percentiles among 491 GP practices representing the proportion of patients in the practice receiving interventions.

	Minimum	10th percentile	25th percentile	Median	75th percentile	90th percentile	Maximum
**Patients receiving any intervention (%)**	0	4	7	12	19	28	91
**Patients receiving advice (%)**	0	0	0	3	9	18	91
**Patients receiving a referral (%)**	0	0	1	3	7	13	50
**Patients receiving anti-obesity drugs (%)**	0	0	2	4	6	9	33

NB: different practices may occupy centiles for different measures

For peer review only - http://bmjopen.bmj.com/site/about/guidelines.xhtml

DISCUSSION

Summary

Analysis of primary care electronic health records reveals that the use of weight management interventions in primary care for the treatment of overweight and obesity were infrequent between 2005 and 2012. The likelihood of intervention was strongly associated with BMI category. However, 60% of men and 58% of women with morbid obesity did not have any record of receiving weight management in primary care, with higher proportions noted in lower BMI categories. Variation in obesity management between general practices was evident, with many practices not recording any intervention. These results might be a consequence of poor documentation of advice given, but might also indicate a lack of patient access to appropriate body weight management interventions in primary care due to a lack of clinician awareness or confidence in treating obesity. Guidelines on the management of obesity from NICE (3) do not appear to have been successfully implemented into practice.

There was some evidence body weight management was tailored to obesity category with more frequent utilisation of anti-obesity drugs in patients who were in higher obesity categories and advice used more commonly in overweight patients. While BMI category was the strongest predictor of a patient receiving weight management interventions, with rates over 3-times higher in morbid obesity than in overweight, female gender, increasing age, socioeconomic deprivation and co-morbidities tended to be associated with greater use of weight management interventions.

Follow-up values for body weight after a recorded weight management intervention were limited. Monitoring of body weight in primary care is generally opportunistic and depends

For peer review only - http://bmjopen.bmj.com/site/about/guidelines.xhtml

on patients attending the practice and having a weight measurement recorded. However, the relatively high levels of co-morbidity in patients in this cohort, including those that require long-term management such as type 2 diabetes, suggest that consultations are likely to be regular. While follow-up weight measurements did not show any change in weight after intervention; these results are very vulnerable to information bias.

Comparison with the literature

One other UK-based study investigated using of primary care interventions for the treatment of obesity.(9) The Counterweight report identified that 20% of patients received advice, 4% referrals and 2% anti-obesity drugs based on a review of 100 obese patients medical records over an 18-month period in 2000-01. We identified a smaller proportion of patients receiving advice and a higher proportion having a referral or drug prescription over a longer time period. It was not clear how obese participants were selected in the Counterweight study. Other differences between the present study and the Counterweight paper include a larger sample size and inclusion of overweight patients. However, the results suggest that prescribing of anti-obesity drugs has increased in the last 15 years. Increased use of anti-obesity drugs between 1998, when they were first introduced in the UK, and 2005 has been reported elsewhere.(16)

A decline in lifestyle advice and counselling for weight loss given to obese patients over the last 10 years has been also been reported in studies from the US. Reasons behind this reduction, despite increasing obesity levels, include poor recording of advice, lack of time in consultations, pessimism regarding potential success of weight loss attempts and increased use of medications to treat obesity-related risk factors and disease (17, 18) and, perhaps, normalisation of excessive body weight. Although the evidence from the current

For peer review only

For peer review only - http://bmjopen.bmj.com/site/about/guidelines.xhtml

study is not sufficient to conclude that a reduction in advice for weight management has occurred, some of the explanations attributed to lowered rates in the US are likely to be applicable in the UK.

Strengths and limitations

This study design had the advantage of a large population-based sample taken from different regions of the UK. However, it is likely that not all weight management interventions, particularly lifestyle advice, were captured in the electronic health record. Brief advice may be given to patients but not recorded by clinicians, which could have led to an underestimation of intervention rates. This is less likely to be an issue with referrals and drug prescribing. Furthermore, the patients included in this sample were selected on the basis of having a BMI record indicating that they were overweight or obese. This may have introduced a selection bias as these patients have been identified as having a weight problem by a clinician. Patients who are obese but do not have a record of weight status in their medical record may or may not be receiving weight management interventions differently from those who have been diagnosed.

Implications for practice and future research

The results of this study suggest that primary-care interventions given to patients with the aim of reducing weight are under-utilised, and that follow-up to determine their success is poor. It is possible that rates have been under-estimated through a lack of formal recording in medical records. However, the growing burden of obesity on primary healthcare services and lack of long-term follow-up on the effectiveness of these treatments supports the use of structured recording of interventions for weight management and subsequent follow-up. This is particularly true given the heterogeneity of results from weight loss

For peer review only - http://bmjopen.bmj.com/site/about/guidelines.xhtml

studies included in reviews of the effectiveness of primary-care interventions for obesity and the need for further evidence specific to patient sub-groups e.g. those with co-morbidities (8, 19, 20). Primary care referrals to commercial weight loss programmes have been found to be effective in trials.(21-22) Although this type of referral wasn't included in the present study, an analysis using primary care data could be valuable. Data in CPRD are not specific enough to permit this at present. Consistency of public health messages on the health risks associated with obesity should be promoted in primary care where clinicians have the opportunity to reach a large number of patients and utilise preventive as well as reactive treatment strategies.

Study funding: This study was supported by the UK National Prevention Research Initiative whose funding partners include the Alzheimer's Research Trust; Alzheimer's Society; Biotechnology and Biological Sciences Research Council; British Heart Foundation; Cancer Research UK; Chief Scientist Office, Scottish Government Health Directorate; Department of Health; Diabetes UK; Economic and Social Research Council; Engineering and Physical Sciences Research Council; Health & Social Care Research & Development Office for Northern Ireland; Medical Research Council; The Stroke Association; Welsh Assembly Government; and World Cancer Research Fund. The views expressed are those of the authors alone.

Competing interests: None declared

Contributions: HB & MG designed the study. TP & MG advised on the conduct of the data analysis. HB conducted the analysis and drafted the paper. MG & TP contributed to interpretation of the results. All authors commented and approved the manuscript. HB is guarantor.

For peer review only - http://bmjopen.bmj.com/site/about/guidelines.xhtml

For peer review only

REFERENCES

1. Lopez AD, Mathers CD, Ezzati M, editors. Global burden of disease and risk factors. Washington: The World Bank & Oxford University Press; 2006.

2. Moody A. Chapter 10: Adult anthropometric measures, overweight and obesity. London, UK: Health and Social Care Information Centre, 2013.

3. National Institute for Health and Care Excellence. Obesity: Identification, assessment and management of overweight and obesity in children, young people and adults (draft for consultation). London, UK: National Institute for Health and Care Excellence, 2014 Contract No.: CG43.

4. Ara R, Blake L, Gray L, et al. What is the clinical effectiveness and cost-effectiveness of using drugs in treating obese patients in primary care? A systematic review. Health Technology Assessment. 2012;16(5).

5. Avenell A, Broom J, Brown TJ, et al. Systematic review of the long-term effects and economic consequences of treatments for obesity and implications for health improvement. Health Technology Assessment. 2004;8(21).

6. Picot J, Jones J, Colquitt J, et al. Weight Loss Surgery for Mild to Moderate Obesity: A Systematic Review and Economic Evaluation. Obesity Surgery. 2012;22(9):1496-506.

7. Picot J, Jones J, Colquitt JL, et al. The clinical effectiveness and cost-effectiveness of bariatric (weight loss) surgery for obesity: a systematic review and economic evaluation. Health Technology Assessment. 2009;13(41):215-357.

8. Booth HP, Prevost AT, Wright AJ, et al. (in press) Effectiveness of behavioral weight loss interventions delivered in a primary care setting: A systematic review and meta-analysis. Family Practice. 2014.

9. Counterweight Project Team. Current approaches to obesity management in UK primary care: the Counterweight programme. J Hum Nutr Diet. 2004;17:7.

For peer review only - http://bmjopen.bmj.com/site/about/guidelines.xhtml

10. Department of Health. Healthy Lives, Healthy People: a call to action on obesity. London: HM Government, 2011 Contract No.: 16166.

11. Williams T, van Staa T, Puri S, et al. Recent advances in the utility and use of the General Practice Research Database as an example of a UK Primary Care Data resource. Therapeutic Advances in Drug Safety. 2012;3(2):89-99.

12. Medicines and Healthcare Products Regulatory Agency. Suspension of sibutramine (Reductil) 2010 [19/03/2014]. Available from: http://www.mhra.gov.uk/NewsCentre/CON068470.

13. Medicines and Healthcare Products Regulatory Agency. Rimonabant: European suspension of marketing authorisation 2008 [cited 26/08/2014]. Available from: http://www.mhra.gov.uk/Safetyinformation/DrugSafetyUpdate/CON087927.

14. Kang JG, Park C-Y. Anti-Obesity Drugs: A Review about Their Effects and Safety. Diabetes Metab J. 2012;36(1):13–25.

15. Cleves M, StataCorp. Analysis of multiple failure-time data with Stata 2009 [cited 10/09/2014]. Available from: http://www.stata.com/support/faqs/statistics/multiple-failure-time-data/.

16. Srishanmuganathan J, Patel H, Car J, Majeed A. National trends in the use and costs of anti-obesity medications in England 1998–2005. J Public Health. 2007;29(2):199-202.

17. Yates EA, Macpherson AK, Kuk JL. Secular Trends in the Diagnosis and Treatment of Obesity Among US Adults in the Primary Care Setting. Obesity. 2012;20(9):1909-14.

18. McAlpine DDW, A R. Trends in Obesity-Related Counseling in Primary Care: 1995–2004. Medical Care. 2007;45(4):322-9.

19. Tsai AG, Wadden TA. Treatment of obesity in primary care practice in the United States: A systematic review. J Gen Intern Med. 2009;24(9):1073-9.

For peer review only

20. LeBlanc ES, O'Connor E, Whitlock EP, et al. Effectiveness of Primary Care–Relevant Treatments for Obesity in Adults: A Systematic Evidence Review for the U.S. Preventive Services Task Force. Ann Int Med. 2011;155(7):434-47.

21. Jebb SA, Ahern AL, Olson AD, et al. Primary care referral to a commercial provider for weight loss treatment versus standard care: a randomised controlled trial. The Lancet. 2011;378(9801):1485-92.

22. Dixon KJL, Shcherba S, Kipping RR. Weight loss from three commercial providers of NHS primary care slimming on referral in North Somerset: service evaluation. J Public Health. 2012;34(4):555-61.

For peer review only

For peer review only - http://bmjopen.bmj.com/site/about/guidelines.xhtml

Access to weight reduction interventions for overweight and obese patients in UK primary care. Population-based cohort study

Running head: Obesity management in primary care

Article category: Original research

Authors:

Helen P. Booth[1], BSc MSc

A. Toby Prevost[1], BSc MSc PhD

Martin C. Gulliford[1], FFPH FRCP

[1]Department of Primary Care and Public Health Sciences, King's College London, UK

Corresponding author:

Miss Helen P. Booth, Research Associate, Department of Primary Care and Public Health Sciences, King's College London, 7th Floor Capital House, 42 Weston Street, London,

SE1 3QD. Tel: 020 7848 6104. Fax: 020 7848 6620 Email: helen.booth@kcl.ac.uk

References: 21

Tables: 5

Figures: 0

Word count: 3,403

Abstract word count: 271

Supplementary files: 0

Key words: Obesity, Overweight, Primary health care, General practice

For peer review only - http://bmjopen.bmj.com/site/about/guidelines.xhtml

For peer review only

ABSTRACT

Objectives

To investigate access to weight management interventions for overweight and obese patients in primary care

Setting

UK primary care electronic health records

Participants

A cohort of 91,413 overweight and obese patients aged 30 to 100 years was sampled from the Clinical Practice Research Datalink (CPRD). Patients with body mass index (BMI) values ≥25 kg/m^2 recorded between 2005 and 2012 were included. BMI values were categorised using World Health Organization (WHO) criteria.

Interventions

Interventions for body weight management, including advice, referrals and prescription of anti-obesity drugs, were evaluated.

Primary and secondary outcome measures

The rate of body weight management interventions and time to intervention were the main outcomes

Results

Data were analysed for 91,413 patients, mean age 56 years, including 55,094 (60%) overweight and 36,319 (40%) obese, including 4,099 (5%) with morbid obesity. During the study period 90% of overweight patients had no weight management intervention recorded. Intervention was more frequent among obese patients, but 59% of patients with morbid obesity had no intervention recorded. Rates of

For peer review only - http://bmjopen.bmj.com/site/about/guidelines.xhtml

intervention increased with BMI category. In morbid obesity, rates of intervention per 1,000 patient years were: advice, 60.2 (95% CI 51.8 to 70.4); referral, 75.7 (95% CI 69.5 to 82.6); and anti-obesity drugs 89.9 (95% CI 85.0 to 95.2). Weight management interventions were more often accessed by women, older patients, those with co-morbidity and those in deprivation. Follow-up of body weight subsequent to interventions was infrequent.

Conclusions

Limited evidence of weight management interventions in primary care electronic health records may result from poor recording of advice given, but may indicate a lack of patient access to appropriate body weight management interventions in primary care.

ARTICLE SUMMARY

Strengths and limitations of this study

- This study uses primary care electronic health records to investigate the use of weight management interventions in overweight and obese patients
- Lifestyle advice was the most commonly-used intervention in all but morbidly obese patients, where anti-obesity drugs were more frequent
- Patients had to have a BMI value recorded to be included in the study
- Clinicians may be giving advice for weight management but not recording it

For peer review only - http://bmjopen.bmj.com/site/about/guidelines.xhtml

BACKGROUND

Obesity is a leading cause of premature morbidity and mortality worldwide.(1) In the UK, a quarter of adults are obese and up to two thirds are overweight.(2) Primary care represents an important setting in which obese patients may access weight loss interventions. The main strategies for treating obesity are provision of lifestyle advice, referral for weight management, prescription of anti-obesity drugs and, in severe cases, referral for bariatric surgery. Clinical guidelines recommend a stepped approach to weight management depending on the severity of a patient's obesity and whether they have weight-related co-morbidities, with more intensive interventions offered as appropriate. Interventions should be agreed between the clinician and patient, and offered in conjunction with long-term follow-up and continuing care.(3)

Several studies have evaluated the effectiveness of primary care interventions for weight management in obesity, (4-8) but few studies have evaluated how overweight and obese patients are managed in primary care. A survey using data collected in 2000/1, before the UK national guidelines on obesity management were published,(9) found that a fifth of obese patients were offered dietary counselling, less than 5% a referral and 2% anti-obesity medications over an 18 month period. There is no more recent information on the use of interventions for the management of obesity in primary care.

Access to appropriate weight management interventions for overweight and obese patients in primary care is of increasing importance in the context of a national objective to establish a downward trend in obesity among UK adults by 2020.(10)

For peer review only - http://bmjopen.bmj.com/site/about/guidelines.xhtml

For peer review only

This study aimed to evaluate access in terms of recording and utilisation of weight management interventions for overweight and obesity using primary care electronic health records. Interventions were classified as lifestyle advice, referrals for weight management and prescription of anti-obesity drugs.

For peer review only

For peer review only - http://bmjopen.bmj.com/site/about/guidelines.xhtml

METHODS

Data source and cohort definition

A cohort of patients was selected from the Clinical Practice Research Datalink (CPRD), a database of longitudinal patient electronic medical records from UK primary care. CPRD is the largest primary care database in the world, and represents over 5% of the UK population with about 680 practices currently contributing research quality data.(11) The initial cohort was selected as part of a larger project, and comprised a random sample of approximately 300,000 patients who were registered for at least 12 months with a general practice contributing data to CPRD between the 1st January 2005 and 30th April 2012. Equal numbers of men and women were drawn from each year of the study without replacement. Patients were selected who had a body mass index (BMI) record indicating overweight or obesity during the study period. The study was approved by the CPRD Independent Scientific Advisory Committee (ISAC 07_054 and 14_056).

Exposure and outcome definitions

BMI was categorised using the World Health Organization (WHO) categories: overweight (BMI 25-29.9kg/m^2), obese (BMI 30-34.9kg/m^2), severe obesity (BMI 35.0 to 39.9kg/m^2), morbid obesity (BMI≥40kg/m^2). Medical diagnoses of obesity in the medical record were also noted based on the presence of diagnostic codes. Morbidity status was ascertained based on the presence of eleven common conditions associated with obesity, including: type 2 diabetes, coronary heart disease (CHD), stroke, depression, osteoarthritis, back pain, joint problems, cancer, gallbladder disease, asthma and sleep apnoea. Smoking status and socioeconomic

For peer review only - http://bmjopen.bmj.com/site/about/guidelines.xhtml

deprivation were also included as exposures. Socioeconomic deprivation was classified into quintiles using the Index of Multiple Deprivation rank based on patient postcode. Data on deprivation were only available for patients registered at English practices.

Interventions for the management of body weight were identified using medical codes recorded in clinical and referral records, recorded health promotion advice, and prescriptions for anti-obesity drugs. For analysis, weight management interventions were classified into lifestyle advice, referrals for weight management and prescription of anti-obesity drugs. Advice included codes relating to dieting, exercise and weight loss. Relevant referrals included those to community and hospital dieticians, for exercise therapy and for weight management programmes. Prescriptions for three different anti-obesity drugs were included; two of these, sibutramine and rimonabant, have been removed from the UK market because of safety concerns.(12, 13) However, these drugs were in use during the time period investigated and so have been considered in this analysis. The only anti-obesity drug currently licensed in the UK, orlistat, was introduced in 1998 and has been available over the counter as well as by prescription since 2009.(14) Multiple prescriptions of anti-obesity drugs were considered to be a part of the same course of treatment if there was less than 6 months between prescriptions.

Analysis

Person time was analysed following the index date; the first BMI record for overweight or obesity after the 1st January 2005. Patient baseline characteristics were tabulated. The proportion of patients who received weight management

For peer review only - http://bmjopen.bmj.com/site/about/guidelines.xhtml

interventions over the study period was evaluated by BMI category. Time-to-event analysis was used to calculate the rate of intervention utilisation by BMI category and to investigate variables associated with intervention using a multiple-failure multivariable Cox proportional hazards regression model with unordered events.(15) Variation in the use of weight management interventions by GP practice was investigated by calculating the proportion of patients receiving any intervention in the year following the index date. These data were then presented as percentiles of the distribution for all practices. Change in weight from baseline after the implementation of each type of intervention was calculated for up to 5 years of follow-up.

For peer review only

For peer review only - http://bmjopen.bmj.com/site/about/guidelines.xhtml

RESULTS

Of the 300,006 patients in the cohort, 134,697 (45%) had an eligible BMI record. After patients with BMIs lower than 25kg/m^2 were removed, data were analysed for 91,413 patients, with mean age 56 years, including 55,094 (60%) overweight and 36,319 (40%).or obese, including 4,099 (5%) with morbid obesity. Mean age in men and women was 56 years. Patient characteristics on entry to the study are presented in Table 1. At the index date (date of the first relevant BMI record) most patients were overweight (63.9% of men and 56.2% of women); 2.9% of men and 6.3% of women were morbidly obese. A diagnostic code for obesity was recorded for 3.9% of male patients and 6.5% of females. A higher proportion of women were non-smokers, while men were more likely to be former or current smokers.

For peer review only

Table 1: Characteristics of overweight and obese patients. Figures are frequencies (column percent) unless stated otherwise.

	Men (48,413)	Women (43,000)
**Mean age (SD)**	55.6 (13.9)	56.4 (15.0)
**Body mass index (BMI) category (Kg/m2)**		
Overweight (BMI 25-29.9)	30,950 (63.9)	24,144 (56.2)
Obese (BMI 30-34.9)	12,711 (26.3)	11,364 (26.4)
Severe obesity (BMI 35-39.9)	3,368 (7.0)	4,777 (11.1)
Morbid obesity (BMI ≥40)	1,384 (2.9)	2,715 (6.3)
**Medical code for obesity**	1,876 (3.9)	2,810 (6.5)
**Number of morbidities**		
0	14,810 (30.6)	9,635 (22.4)
1	14,988 (31.0)	11,919 (27.7)
2	10,323 (21.3)	10,237 (23.8)
3 or more	8,292 (17.1)	11,209 (26.1)
**Smoking status**		
Non-smoker	17,415 (36.0)	20,602 (47.9)
Ex-smoker	15,188 (31.4)	9,916 (23.1)
Current smoker	9,359 (19.3)	7,448 (17.3)
Missing smoking status	6,451 (13.3)	5,034 (11.7)
**Index of Multiple Deprivation (IMD) quintile**		
1 – least deprived	11,490 (23.7)	9,229 (21.5)
2	10,850 (22.4)	9,275 (21.6)
3	8,858 (18.3)	7,896 (18.4)
4	7,859 (16.2)	7,413 (17.2)
5 – most deprived	6,310 (13.0)	6,304 (14.7)
Missing IMD	3,046 (6.3)	2,883 (6.7)

For peer review only

For peer review only - http://bmjopen.bmj.com/site/about/guidelines.xhtml

The majority of patients did not receive a weight management intervention during the study period. The proportion of patients by BMI category with each type of intervention recorded on their medical record is given in Table 2. In patients with morbid obesity, 60.0% of men and 58.1% of women had no record of weight management during the 7 years of the study. In patients with non-severe obesity (BMI 30-34.9kg/m^2) the figures were 84.2% and 80.2% respectively. The proportion of patients who received an intervention increased with each additional BMI category. Advice was the most commonly recorded intervention in overweight and obese patients and severely obese men. Drug interventions were the most frequently recorded intervention in morbidly obese patients and severely obese women.

Table 2: Patients who received, or did not receive, a weight management intervention over the study period by gender and BMI category. Figures are frequencies (row percent).

BMI category	Total	Advice	Referral	Drugs	No treatment
		**Men**			
Overweight (BMI 25-29.9)	30,950	1,805 (5.8)	913 (2.9)	86 (0.3)	28,282 (91.4)
Obese (BMI 30-34.9)	12,711	1,129 (8.9)	762 (6.0)	313 (2.5)	10,697 (84.2)
Severe obesity (BMI 35-39.9)	3,368	363 (10.8)	349 (10.4)	333 (9.9)	2,499 (74.2)
Morbid obesity (BMI ≥40)	1,384	168 (12.1)	239 (17.3)	322 (23.3)	831 (60.0)
		**Women**			
Overweight (BMI 25-29.9)	24,144	1,331 (5.5)	762 (3.2)	451 (1.9)	21,794 (90.3)
Obese (BMI 30-34.9)	11,364	925 (8.1)	740 (6.5)	889 (7.8)	9,116 (80.2)
Severe obesity (BMI 35-39.9)	4,777	462 (9.7)	445 (9.3)	671 (14.0)	3,460 (72.4)
Morbid obesity (BMI ≥40)	2,715	284 (10.5)	479 (17.6)	724 (26.7)	1,578 (58.1)

For peer review only - http://bmjopen.bmj.com/site/about/guidelines.xhtml

Rates of intervention are presented in Table 3. Overall, the recorded rates of intervention were highest for advice at 30.3 (95% CI 29.3 to 31.4) per 1,000 person-years. The rate of each intervention type increased in higher BMI categories. The rate of advice was 22.6 (21.6 to 23.8) per 1,000 in overweight patients, and highest at 60.2 (51.8 to 70.4) per 1,000 in morbidly obese patients. In overweight patients, advice was the most commonly used intervention, whereas drug prescription was the most common in morbidly obese patients.

Table 3: Rate of obesity management intervention by body mass index (BMI) category (per 1000 patient years), based on records of advice, referral or obesity drug prescription.

	Advice	Referral	Drugs
**Overall rate**	30.3 (29.3 to 31.4)	20.0 (19.3 to 20.8)	13.9 (13.5 to 14.4)
**Overweight (BMI 25-29.9)**	22.6 (21.6 to 23.8)	11.2 (10.5 to 11.9)	2.9 (2.6 to 3.2)
**Obese (BMI 30-34.9)**	36.4 (34.4 to 38.6)	23.7 (22.3 to 25.2)	15.7 (14.7 to 16.7)
**Severe obesity (BMI 35-39.9)**	47.2 (42.7 to 52.3)	38.4 (35.4 to 41.7)	41.5 (39.0 to 44.1)
**Morbid obesity (BMI ≥40)**	60.2 (51.8 to 70.4)	75.7 (69.5 to 82.6)	89.9 (85.0 to 95.2)

For peer review only - http://bmjopen.bmj.com/site/about/guidelines.xhtml

The multivariable analysis identified BMI category as the strongest predictor of weight-loss intervention, with a hazard ratio (HR) of 1.68 (95% CI 1.60 to 1.76) for obesity and 3.67 (95% CI 3.45 to 3.92) for morbid obesity (Table 4). Increasing age, type 2 diabetes and depression tended to be associated with receiving a weight loss intervention. Female gender, being a former smoker and socioeconomic deprivation were associated with treatment for overweight and obesity.

Table 4: Cox proportional hazards model investigating time to multiple weight management interventions after a record of overweight or obesity.

	Patients receiving weight management intervention (n)	Total patients (N)	Hazard ratio	95% confidence interval	P value
**Age (decades)**	-	-	1.42	1.27 to 1.58	<0.001
**Age squared**	-	-	0.97	0.96 to 0.98	<0.001
**Gender**					
Male	6,104	48,413	1.00	-	-
Female	7,054	43,000	1.14	1.10 to 1.19	<0.001
**Body mass index (BMI) category***					
Overweight (BMI 25-29.9)	5,019	50,075	1.00	-	-
Obese (BMI 30-34.9)	4,263	19,812	1.68	1.60 to 1.76	<0.001
Severe obesity (BMI 35-39.9)	2,186	5,959	2.36	2.23 to 2.50	<0.001
Morbid obesity (BMI ≥40)	1,690	2,409	3.67	3.45 to 3.91	<0.001
**Smoking status**					
Non-smoker	5,441	32,576	1.00	-	-
Former smoker	3,962	24,142	1.11	1.06 to 1.16	<0.001
Current smoker	2,530	14,277	0.99	0.94 to 1.05	0.823
Missing smoking status	1,225	10,260	0.82	0.77 to 0.88	<0.001
**Index of Multiple Deprivation quintile**					
1 - least deprived	2,564	18,155	1.00	-	-
2	2,490	17,635	0.94	0.88 to 1.00	0.054
3	2,511	14,243	1.20	1.12 to 1.29	<0.001
4	2,413	12,859	1.13	1.06 to 1.21	<0.001
5 – most deprived	2,277	10,337	1.24	1.15 to 1.32	<0.001
Missing IMD	903	5,026	1.04	0.95 to 1.13	0.395
**Coronary heart disease (CHD)**	1,993	9,669	1.24	1.16 to 1.31	<0.001
**Stroke**	535	2,603	1.09	0.98 to 1.21	0.116
**Type 2 diabetes**	4,401	12,884	1.83	1.75 to 1.92	<0.001
**Depression**	6,385	31,573	1.33	1.28 to 1.39	<0.001

* BMI group at baseline. Patients could change BMI category in the analysis so intervention may have been delivered when patients had changed BMI category

For peer review only - http://bmjopen.bmj.com/site/about/guidelines.xhtml

For peer review only

There was substantial variation between practices in the recording of obesity management interventions (see Table 5). The median proportion of obese and overweight patients receiving a weight management intervention during the study was 12% (Interquartile range (IQR) 7 to 19). A maximum of 91% overweight or obese patients in a practice had an intervention recorded. Follow-up measurements of body weight after intervention were most frequent in patients who had a referral, with 34.1% of patients having a weight measurement in the first year. In contrast, 20.7% of patients had a follow-up weight measurement in the first year after advice and 24.3% after a drug prescription. No trend in weight change was observed in patients up to five years after any of the three intervention types investigated.

Table 5: Use of weight management interventions in general practices. Figures are percentiles among 491 GP practices representing the proportion of patients in the practice receiving interventions.

	Minimum	10th percentile	25th percentile	Median	75th percentile	90th percentile	Maximum
**Patients receiving any intervention (%)**	0	4	7	12	19	28	91
**Patients receiving advice (%)**	0	0	0	3	9	18	91
**Patients receiving a referral (%)**	0	0	1	3	7	13	50
**Patients receiving anti-obesity drugs (%)**	0	0	2	4	6	9	33

NB: different practices may occupy centiles for different measures

For peer review only - http://bmjopen.bmj.com/site/about/guidelines.xhtml

DISCUSSION

Summary

Analysis of primary care electronic health records reveals that the use of weight management interventions in primary care for the treatment of overweight and obesity were infrequent between 2005 and 2012. The likelihood of intervention was strongly associated with BMI category. However, 60% of men and 58% of women with morbid obesity did not have any record of receiving weight management in primary care, with higher proportions noted in lower BMI categories. Variation in obesity management between general practices was evident, with many practices not recording any intervention. These results might be a consequence of poor documentation of advice given, but might also indicate a lack of patient access to appropriate body weight management interventions in primary care due to a lack of clinician awareness or confidence in treating obesity. Guidelines on the management of obesity from NICE (3) do not appear to have been successfully implemented into practice.

There was some evidence body weight management was tailored to obesity category with more frequent utilisation of anti-obesity drugs in patients who were in higher obesity categories and advice used more commonly in overweight patients. While BMI category was the strongest predictor of a patient receiving weight management interventions, with rates over 3-times higher in morbid obesity than in overweight, female gender, increasing age, socioeconomic deprivation and co-morbidities tended to be associated with greater use of weight management interventions.

Follow-up values for body weight after a recorded weight management intervention were limited. Monitoring of body weight in primary care is generally opportunistic and depends

For peer review only - http://bmjopen.bmj.com/site/about/guidelines.xhtml

on patients attending the practice and having a weight measurement recorded. However, the relatively high levels of co-morbidity in patients in this cohort, including those that require long-term management such as type 2 diabetes, suggest that consultations are likely to be regular. While follow-up weight measurements did not show any change in weight after intervention; these results are very vulnerable to information bias.

Comparison with the literature

One other UK-based study investigated using of primary care interventions for the treatment of obesity.(9) The Counterweight report identified that 20% of patients received advice, 4% referrals and 2% anti-obesity drugs based on a review of 100 obese patients medical records over an 18-month period in 2000-01. We identified a smaller proportion of patients receiving advice and a higher proportion having a referral or drug prescription over a longer time period. It was not clear how obese participants were selected in the Counterweight study. Other differences between the present study and the Counterweight paper include a larger sample size and inclusion of overweight patients. However, the results suggest that prescribing of anti-obesity drugs has increased in the last 15 years. Increased use of anti-obesity drugs between 1998, when they were first introduced in the UK, and 2005 has been reported elsewhere.(16)

A decline in lifestyle advice and counselling for weight loss given to obese patients over the last 10 years has been also been reported in studies from the US. Reasons behind this reduction, despite increasing obesity levels, include poor recording of advice, lack of time in consultations, pessimism regarding potential success of weight loss attempts and increased use of medications to treat obesity-related risk factors and disease (17, 18) and, perhaps, normalisation of excessive body weight. Although the evidence from the current

For peer review only

For peer review only - http://bmjopen.bmj.com/site/about/guidelines.xhtml

study is not sufficient to conclude that a reduction in advice for weight management has occurred, some of the explanations attributed to lowered rates in the US are likely to be applicable in the UK.

Strengths and limitations

This study design had the advantage of a large population-based sample taken from different regions of the UK. However, it is likely that not all weight management interventions, particularly lifestyle advice, were captured in the electronic health record. Brief advice may be given to patients but not recorded by clinicians, which could have led to an underestimation of intervention rates. This is less likely to be an issue with referrals and drug prescribing. Furthermore, the patients included in this sample were selected on the basis of having a BMI record indicating that they were overweight or obese. This may have introduced a selection bias as these patients have been identified as having a weight problem by a clinician. Patients who are obese but do not have a record of weight status in their medical record may or may not be receiving weight management interventions differently from those who have been diagnosed.

Implications for practice and future research

The results of this study suggest that primary-care interventions given to patients with the aim of reducing weight are under-utilised, and that follow-up to determine their success is poor. It is possible that rates have been under-estimated through a lack of formal recording in medical records. However, the growing burden of obesity on primary healthcare services and lack of long-term follow-up on the effectiveness of these treatments supports the use of structured recording of interventions for weight management and subsequent follow-up. This is particularly true given the heterogeneity of results from weight loss

For peer review only

For peer review only - http://bmjopen.bmj.com/site/about/guidelines.xhtml

studies included in reviews of the effectiveness of primary-care interventions for obesity and the need for further evidence specific to patient sub-groups e.g. those with co-morbidities (8, 19, 20). Primary care referrals to commercial weight loss programmes have been found to be effective in trials.(21-22) Although this type of referral wasn't included in the present study, an analysis using primary care data could be valuable. Data in CPRD are not specific enough to permit this at present. Consistency of public health messages on the health risks associated with obesity should be promoted in primary care where clinicians have the opportunity to reach a large number of patients and utilise preventive as well as reactive treatment strategies.

Study funding: This study was supported by the UK National Prevention Research Initiative whose funding partners include the Alzheimer's Research Trust; Alzheimer's Society; Biotechnology and Biological Sciences Research Council; British Heart Foundation; Cancer Research UK; Chief Scientist Office, Scottish Government Health Directorate; Department of Health; Diabetes UK; Economic and Social Research Council; Engineering and Physical Sciences Research Council; Health & Social Care Research & Development Office for Northern Ireland; Medical Research Council; The Stroke Association; Welsh Assembly Government; and World Cancer Research Fund. The views expressed are those of the authors alone.

Competing interests: None declared

Contributions: HB & MG designed the study. TP & MG advised on the conduct of the data analysis. HB conducted the analysis and drafted the paper. MG & TP contributed to interpretation of the results. All authors commented and approved the manuscript. HB is guarantor.

For peer review only - http://bmjopen.bmj.com/site/about/guidelines.xhtml

For peer review only

REFERENCES

1. Lopez AD, Mathers CD, Ezzati M, editors. Global burden of disease and risk factors. Washington: The World Bank & Oxford University Press; 2006.

2. Moody A. Chapter 10: Adult anthropometric measures, overweight and obesity. London, UK: Health and Social Care Information Centre, 2013.

3. National Institute for Health and Care Excellence. Obesity: Identification, assessment and management of overweight and obesity in children, young people and adults (draft for consultation). London, UK: National Institute for Health and Care Excellence, 2014 Contract No.: CG43.

4. Ara R, Blake L, Gray L, et al. What is the clinical effectiveness and cost-effectiveness of using drugs in treating obese patients in primary care? A systematic review. Health Technology Assessment. 2012;16(5).

5. Avenell A, Broom J, Brown TJ, et al. Systematic review of the long-term effects and economic consequences of treatments for obesity and implications for health improvement. Health Technology Assessment. 2004;8(21).

6. Picot J, Jones J, Colquitt J, et al. Weight Loss Surgery for Mild to Moderate Obesity: A Systematic Review and Economic Evaluation. Obesity Surgery. 2012;22(9):1496-506.

7. Picot J, Jones J, Colquitt JL, et al. The clinical effectiveness and cost-effectiveness of bariatric (weight loss) surgery for obesity: a systematic review and economic evaluation. Health Technology Assessment. 2009;13(41):215-357.

8. Booth HP, Prevost AT, Wright AJ, et al. (in press) Effectiveness of behavioral weight loss interventions delivered in a primary care setting: A systematic review and meta-analysis. Family Practice. 2014.

9. Counterweight Project Team. Current approaches to obesity management in UK primary care: the Counterweight programme. J Hum Nutr Diet. 2004;17:7.

For peer review only - http://bmjopen.bmj.com/site/about/guidelines.xhtml

10. Department of Health. Healthy Lives, Healthy People: a call to action on obesity. London: HM Government, 2011 Contract No.: 16166.

11. Williams T, van Staa T, Puri S, et al. Recent advances in the utility and use of the General Practice Research Database as an example of a UK Primary Care Data resource. Therapeutic Advances in Drug Safety. 2012;3(2):89-99.

12. Medicines and Healthcare Products Regulatory Agency. Suspension of sibutramine (Reductil) 2010 [19/03/2014]. Available from: http://www.mhra.gov.uk/NewsCentre/CON068470.

13. Medicines and Healthcare Products Regulatory Agency. Rimonabant: European suspension of marketing authorisation 2008 [cited 26/08/2014]. Available from: http://www.mhra.gov.uk/Safetyinformation/DrugSafetyUpdate/CON087927.

14. Kang JG, Park C-Y. Anti-Obesity Drugs: A Review about Their Effects and Safety. Diabetes Metab J. 2012;36(1):13–25.

15. Cleves M, StataCorp. Analysis of multiple failure-time data with Stata 2009 [cited 10/09/2014]. Available from: http://www.stata.com/support/faqs/statistics/multiple-failure-time-data/.

16. Srishanmuganathan J, Patel H, Car J, Majeed A. National trends in the use and costs of anti-obesity medications in England 1998–2005. J Public Health. 2007;29(2):199-202.

17. Yates EA, Macpherson AK, Kuk JL. Secular Trends in the Diagnosis and Treatment of Obesity Among US Adults in the Primary Care Setting. Obesity. 2012;20(9):1909-14.

18. McAlpine DDW, A R. Trends in Obesity-Related Counseling in Primary Care: 1995–2004. Medical Care. 2007;45(4):322-9.

19. Tsai AG, Wadden TA. Treatment of obesity in primary care practice in the United States: A systematic review. J Gen Intern Med. 2009;24(9):1073-9.

For peer review only - http://bmjopen.bmj.com/site/about/guidelines.xhtml

20. LeBlanc ES, O'Connor E, Whitlock EP, et al. Effectiveness of Primary Care–Relevant Treatments for Obesity in Adults: A Systematic Evidence Review for the U.S. Preventive Services Task Force. Ann Int Med. 2011;155(7):434-47.

21. Jebb SA, Ahern AL, Olson AD, et al. Primary care referral to a commercial provider for weight loss treatment versus standard care: a randomised controlled trial. The Lancet. 2011;378(9801):1485-92.

22. Dixon KJL, Shcherba S, Kipping RR. Weight loss from three commercial providers of NHS primary care slimming on referral in North Somerset: service evaluation. J Public Health. 2012;34(4):555-61.

For peer review only

STROBE Statement—checklist of items that should be included in reports of observational studies

	Item No	Recommendation
**Title and abstract**	1	(*a*) Indicate the study's design with a commonly used term in the title or the abstract [p1]
		(*b*) Provide in the abstract an informative and balanced summary of what was done and what was found [p2]
**Introduction**		
Background/rationale	2	Explain the scientific background and rationale for the investigation being reported [p4, 2nd para]
Objectives	3	State specific objectives, including any prespecified hypotheses [abstract, p2 and p5]
**Methods**		
Study design	4	Present key elements of study design early in the paper [p6]
Setting	5	Describe the setting, locations, and relevant dates, including periods of recruitment, exposure, follow-up, and data collection [p6]
Participants	6	(*a*) *Cohort study*—Give the eligibility criteria, and the sources and methods of selection of participants. Describe methods of follow-up [p6]<br>*Case-control study*—Give the eligibility criteria, and the sources and methods of case ascertainment and control selection. Give the rationale for the choice of cases and controls<br>*Cross-sectional study*—Give the eligibility criteria, and the sources and methods of selection of participants
		(*b*) *Cohort study*—For matched studies, give matching criteria and number of exposed and unexposed<br>*Case-control study*—For matched studies, give matching criteria and the number of controls per case
Variables	7	Clearly define all outcomes, exposures, predictors, potential confounders, and effect modifiers. Give diagnostic criteria, if applicable [p6 – exposure and outcome definitions]
Data sources/ measurement	8*	For each variable of interest, give sources of data and details of methods of assessment (measurement). Describe comparability of assessment methods if there is more than one group [p6 –7]
Bias	9	Describe any efforts to address potential sources of bias
Study size	10	Explain how the study size was arrived at
Quantitative variables	11	Explain how quantitative variables were handled in the analyses. If applicable, describe which groupings were chosen and why [p6 – exposure and outcome definitions]
Statistical methods	12	(*a*) Describe all statistical methods, including those used to control for confounding [p7-8]
		(*b*) Describe any methods used to examine subgroups and interactions
		(*c*) Explain how missing data were addressed
		(*d*) *Cohort study*—If applicable, explain how loss to follow-up was addressed<br>*Case-control study*—If applicable, explain how matching of cases and controls was addressed<br>*Cross-sectional study*—If applicable, describe analytical methods taking account of

For peer review only

For peer review only - http://bmjopen.bmj.com/site/about/guidelines.xhtml

sampling strategy

(*e*) Describe any sensitivity analyses

Continued on next page

For peer review only

For peer review only - http://bmjopen.bmj.com/site/about/guidelines.xhtml

**Results**		
Participants	13*	(a) Report numbers of individuals at each stage of study—eg numbers potentially eligible, examined for eligibility, confirmed eligible, included in the study, completing follow-up, and analysed
		(b) Give reasons for non-participation at each stage
		(c) Consider use of a flow diagram
Descriptive data	14*	(a) Give characteristics of study participants (eg demographic, clinical, social) and information on exposures and potential confounders [p9 & Table 1]
		(b) Indicate number of participants with missing data for each variable of interest [Table 1]
		(c) *Cohort study*—Summarise follow-up time (eg, average and total amount)
Outcome data	15*	*Cohort study*—Report numbers of outcome events or summary measures over time [p11-12]
		Case-control study—Report numbers in each exposure category, or summary measures of exposure
		Cross-sectional study—Report numbers of outcome events or summary measures
Main results	16	(*a*) Give unadjusted estimates and, if applicable, confounder-adjusted estimates and their precision (eg, 95% confidence interval). Make clear which confounders were adjusted for and why they were included [throughout results]
		(*b*) Report category boundaries when continuous variables were categorized [p6 – BMI]
		(*c*) If relevant, consider translating estimates of relative risk into absolute risk for a meaningful time period
Other analyses	17	Report other analyses done—eg analyses of subgroups and interactions, and sensitivity analyses
**Discussion**		
Key results	18	Summarise key results with reference to study objectives [p15]
Limitations	19	Discuss limitations of the study, taking into account sources of potential bias or imprecision. Discuss both direction and magnitude of any potential bias [p17]
Interpretation	20	Give a cautious overall interpretation of results considering objectives, limitations, multiplicity of analyses, results from similar studies, and other relevant evidence [p17]
Generalisability	21	Discuss the generalisability (external validity) of the study results [p17]
**Other information**		
Funding	22	Give the source of funding and the role of the funders for the present study and, if applicable, for the original study on which the present article is based [p18]

*Give information separately for cases and controls in case-control studies and, if applicable, for exposed and unexposed groups in cohort and cross-sectional studies.

Note: An Explanation and Elaboration article discusses each checklist item and gives methodological background and published examples of transparent reporting. The STROBE checklist is best used in conjunction with this article (freely available on the Web sites of PLoS Medicine at http://www.plosmedicine.org/, Annals of Internal Medicine at http://www.annals.org/, and Epidemiology at http://www.epidem.com/). Information on the STROBE Initiative is available at www.strobe-statement.org.

For peer review only
